# Organic Solution Advanced Spray-Dried Microparticulate/Nanoparticulate Dry Powders of Lactomorphin for Respiratory Delivery: Physicochemical Characterization, In Vitro Aerosol Dispersion, and Cellular Studies

**DOI:** 10.3390/pharmaceutics13010026

**Published:** 2020-12-25

**Authors:** Wafaa Alabsi, Fahad A. Al-Obeidi, Robin Polt, Heidi M. Mansour

**Affiliations:** 1 Department of Chemistry & Biochemistry, The University of Arizona, Tucson, AZ 85721, USA; alabsi@pharmacy.arizona.edu (W.A.); fahada2@email.arizona.edu (F.A.A.-O.); polt@u.arizona.edu (R.P.); 2 College of Pharmacy, Skaggs Pharmaceutical Sciences Center, The University of Arizona, Tucson, AZ 85721, USA; 3 College of Medicine, Division of Translational & Regenerative Medicine, The University of Arizona, Tucson, AZ 85721, USA

**Keywords:** CNS therapeutics, brain, intranasal delivery, pulmonary delivery, dry powder inhalers (DPIs), human cells, glycopeptides, LogP, microscopy, modeling

## Abstract

The purpose of this study was to formulate Lactomorphin (MMP2200) in its pure state as spray-dried(SD) powders, and with the excipient Trehalose as co-spray-dried(co-SD) powders; for intranasal and deep lung administration with Dry Powder Inhalers (DPI). Lactomorphin is a glycopeptide which was developed for the control of moderate to severe pain. Particles were rationally designed and produced by advanced spray drying particle engineering in a closed mode from a dilute organic solution. Comprehensive physicochemical characterization using different analytical techniques was carried out to analyze the particle size, particle morphology, particle surface morphology, solid-state transitions, crystallinity/non-crystallinity, and residual water content. The particle chemical composition was confirmed using attenuated total reflectance-Fourier-transform infrared (ATR-FTIR), and Confocal Raman Microscopy (CRM) confirmed the particles’ chemical homogeneity. The solubility and Partition coefficient (LogP) of Lactomorphin were determined by the analytical and computational methodology and revealed the hydrophilicity of Lactomorphin. A thermal degradation study was performed by exposing samples of solid-state Lactomorphin to a high temperature (62 °C) combined with zero relative humidity (RH) and to a high temperature (62 °C) combined with a high RH (75%) to evaluate the stability of Lactomorphin under these two different conditions. The solid-state processed particles exhibited excellent aerosol dispersion performance with an FDA-approved human DPI device to reach lower airways. The cell viability resazurin assay showed that Lactomorphin is safe up to 1000 μg/mL on nasal epithelium cells, lung cells, endothelial, and astrocyte brain cells.

## 1. Introduction

**Figure 1:**
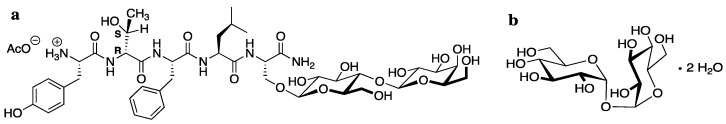
The chemical structures of Lactomorphin/MMP2200 (**a**), and Trehalose dihydrate (**b**).

Chronic pain is a significant health problem throughout the world. Untreated chronic pain can interfere with sleep, limit activities, and decrease overall productivity [1]. Opioid agonists are currently the most widely used analgesic class of drugs to treat pain. They bind to the µ opioid receptor (MOR), which causes the analgesic effects, besides an array of side effects includes respiratory depression, addiction liability, development of tolerance and physical dependence, etc. [1]. As these side effects are mainly mediated through MOR, more interest in targeting the δ opioid receptor (DOR) to produce enough analgesia with low side effects [2,3]. Our group previously reported that the glycopeptide drug candidate Lactomorphin [MMP-2200; H_2_N-Tyr-D-Thr- Gly-Phe-Leu-Ser-(O-β-Lactoside)-CONH_2_, (Figure 1) is a mixed DOR and MOR receptors’ agonist. When administered to rodents *i.v.* or *s.c.* it promoted analgesic effects synergically at these two receptors and produced an improved side effect profile compared with the MOR selective agonist morphine [1]. The comparative in vivo antinociceptive effects of Lactomorphin and morphine by different administration routes including intracerebroventricular, intravenous, and subcutaneous routes [1,4]. The purpose of this study was to design and develop spray-dried (SD) powders of Lactomorphin and co-spray-dried (co-SD) powders of Lactomorphin:Trehalose with favorable physicochemical properties tailored for respiratory delivery as inhaled aerosols. Specifically, delivery to the upper respiratory tract to target the brain through the olfactory route bypassing the blood-brain barrier (BBB), i.e., needle-free nose-to-brain delivery. In addition, delivery to the lower respiratory tract, since the inhalation aerosol drug delivery offers attractive advantages in delivering the drug to the CNS at a low dose while minimizing systemic adverse effects. The lungs contain μ and δ opioid receptors and Lactomorphin is a mixed agonist for these two receptors.

The brain is a unique and complex organ that is highly protected and isolated from the systemic circulation by two major barriers, the BBB and the blood-cerebrospinal fluid barrier (BCSFB) [5]. The BBB’s functional unit includes the capillary endothelial cells, pericytes, and perivascular astrocytes in constant and close contact with the endothelium. These anatomical features and the interaction of these cells support the brain capillary phenotype [5]. The BBB’s endothelial cells are different from the corresponding cells in the periphery in they have minimal pinocytotic activity, increased mitochondrial content, a lack of fenestrations, and the presence of tight junctions [6,7]. The BBB is a barrier for many therapeutics in drug development [8]. The intranasal (IN) route is one of the most promising strategies to deliver drugs non-invasivelyto the brain [8]. Several mechanisms of drug transport through the olfactory epithelium are known and include the transcellular route (i.e., through the cells), the paracellular route (i.e., between the cells), and through the olfactory neuronal cells by intracellular axonal transport [9]. The nasal route of administration has many advantages including low dose, higher local bioavailability in the CNS, bypassing the BBB, shorter time to drug onset, non- invasiveness, and self-administration [10]. Inhalation aerosol delivery via the respiratory tract to target the CNS non-invasively is an alternative route to injectible drug administration and has unique advantages [11,12,13]. The lung is a low metabolic organ compared to the gastrointestinal (GI) tract. It allows rapid and high drug absorption due to the large surface area, the high blood flow, and the absence of first-pass metabolism [11,12]. Nasal delivery is represents a viable strategy for treating CNS diseases [14]. Orally inhaled loxapine (Adasuve^®^) is delivered by a novel handheld device (Staccato^®^ delivery system) to the lungs and was developed for rescue treatment of acute attacks in schizophrenia or bipolar disorder [15]. Inhaled opioids used for pain relief were investigated for several indications such as dyspnea at rest associated with severe heart or lung disease, exercise tolerance, pain after surgery, and the provision of analgesia in general practice [16].

In this study, the organic solution closed-mode spray drying technique was applied to prepare pure Lactomorphin and co-SD-Lactomorphin: Trehalose DPIs. Spray drying is a particle engineering design process mainly used to convert various liquid formulations (aqueous, organic solutions, emulsions, and suspensions) into dry powders. It is a simple, fast, and scalable process used in the chemical, food, and pharmaceutical industries [17,18]. Particle engineering by spray drying can be done by controlling and tailoring spray drying parameters, such as feed solution conditions (i.e., feed type, solvent type, concentration, and feed rate), gas type, inlet/outlet temperatures, and outlet and gas flow rate [19,20]. By optimizing the spray drying parameters, particles can be tailored with optimized particle size, size distribution, particle morphology, and particle surface properties (e.g., surface morphology and interparticulate forces such as van der Waals, electrostatic, and capillary forces) [19,21]. The reduction of residual water in the solid-state particles due to the organic feed solution (no water) enhances aerosol dispersion performance of inhalable powders [22,23]. Comprehensive physicochemical characterization, stability, solubility, lipophilicity, and in vitro aerosol dispersion performance were conducted. In vitro human cell viability studies as a function of Lactomorphin concentration were also carried out.

## 2. Materials and Methods

### 2.1. Materials

Lactomorphin [C_45_H_67_N_7_O_19_; molecular weight (MW): 1010.05 g/mol], shown in Figure 1a (ChemDraw Professional 16.0., CambridgeSoft, Cambridge, MA, USA), was obtained from UCB-Bioproducts-Belgium. Raw D (+)-Trehalose dihydrate (C_12_H_22_O_11_·2H_2_O; MW: 378.32 g/mol) (Figure 1b) was obtained from Acros Organics (Fair Lawn, NJ, USA). Methanol HPLC-grade ACS- certified grade with 99.9% purity was obtained from Fisher Scientific (Fair Lawn, NJ, USA). 2-Propanol HPLC-grade, ACS-certified grade with 99.9% purity was obtained from Fisher Scientific (Fair Lawn, NJ, USA). Ethanol HPLC-grade with 99.9% purity and 1-octanol (ACS reagent grade with purity ≥99.9%) were obtained from Sigma-Aldrich (Milwaukee, WI, USA). AquaStar anhydrous methanol was obtained from EMD Millipore Corporation, an Affiliate of Merck (Dramstadt, Germany). Hydranal^®^-Coulomat AD was obtained from Honeywell Fluka. Water was obtained by Milli-Q P-QOD set-up from Millipore (Fair Lawn, NJ, USA) with a resistivity of 18.1 megaOhm-cm. Ultra-high purity (UHP) nitrogen gas (Cryogenics and gas facility, The University of Arizona, Tucson, AZ, USA) was used. Raw Lactomorphin was stored in sealed glass desiccators over Indicating Drierite/Drierite™ desiccant at −80 °C freezer. Raw D (+)-Trehalose dihydrate was stored in a −20 °C freezer. All materials were used as received.

RPMI 2650 (ATCC^®^CCL-30™) epithelial anaplastic squamous cell carcinoma of the human nasal septum, and Eagle’s Minimum Essential Medium (EMEM) (ATCC^®^ 30-2003™) were used. The media was supplemented with fetal Bovine Serum (FBS) (gibco, Qualified One Shot™) 50 mL, pen-Strep (10,000 Unite/mL penicillin, 10,000 μg/mL streptomycin), and Fungizone (250 μg/mL amphotericin). Human blood-brain barrier hCMEC/D3 Cell Line (Cat. # SCC066) and EndoGRO™-MV Complete Media Kit (Cat. No. SCME004). The media was supplemented with 1 ng/mL FGF-2 (Cat. No.GF003), collagen Type I, Rat Tail (Cat. No. 08-115), and PBS pH7.4 (1X) (Gibco, 10010-023). Cryopreserved cells (single donor) CC-2565 Normal Human Astrocytes (NHA) ≥ 1,000,000 cells (Lonza, Basel, Switzerland), and AGM™ BulletKit™ Kit (CC-3186, Lonza), which contains a 500 mL bottle of ABM™, (CC-3187, Lonza) and AGM™ SingleQuots™ (CC-4123, Lonza), ABM™ (CC-3187, Lonza) Astrocyte basal medium (no growth factors) (500 mL). AGM™ SingleQuots™ (CC-4123, Lonza) Supplements for a complete growth medium, developed especially for NHA (CC-2565, Lonza). For culture and subculture, Trypsin/EDTA (CC-5012, Lonza), Trypsin neutralizing solution (CC-5002, Lonza), and HEPES buffered saline solution (CC-5022, Lonza) were used. H441 lung adenocarcinoma cells (ATCC^®^HTB174^™^), A549 adenocarcinoma human alveolar basal epithelial cells (ATCC^®^ CCL-185^™^), and RPMI Medium 1640(1X) (Gibco, 11875-093) were used.

### 2.2. Methods

#### 2.2.1. Preparation of SD and co-SD Particles by Organic Solution Advanced Spray Drying in Closed Mode

A Büchi B-290 Mini Spray Dryer was used to perform an organic solution advanced spray drying process. The Mini Spray Dryer coupled with a high-performance cyclone in closed mode using UHP dry nitrogen gas as the atomizing drying gas and connected to the B-295 Inert Loop (Büchi Labortechnik AG, Flawil, Switzerland). The stainless steel two-fluid nozzle tip of this instrument was of a diameter 0.7 mm with a 1.5 mm gas cap. (Table 1) lists the spray drying conditions for one and two-component powders and the corresponding outlet temperatures for different formulations. The other spray drying conditions include the aspiration rate (35 m^3^/h at 100% rate), the drying gas atomization rate (670 L/h at 55 mm in height), inlet temperature (150 °C), and Inert loop temperature (−15 °C) all were maintained constant during all the experiments. Four feed pump rates were employed to obtain the SD-Lactomorphin particles using pump rates of 7.5 mL/min (25%) (low pump rate), 15 mL/min (50%) (medium pump rate), 22.5 mL/min (75%) (high pump rate), and 30 mL/min (100%) (high pump rate). The feed solutions were prepared by dissolving Lactomorphin in alcohol (methanol, ethanol, and isopropanol) to make a dilute solution with a concentration of 0.5% *w/v*. For Trehalose and the two-component system, the components were dissolved successively in methanol in rationally selected molar ratios. Only 7.5 mL/min (25%) (low pump rate) and methanol as a solvent were employed to obtain the co-SD-Lactomorphin:Trehalose particles since the produced SD-Lactomorphin of this low pump rate and solvent exhibited the smallest particle size, particle size distribution, and the most smooth and spherical particles, with lowest residual moisture content compared of SD-Lactomorphin powders produced with the other pump rates. All SD and co-SD powders were carefully stored in sealed glass vials stored in sealed glass desiccators indicating Drierite/Drierite™ desiccant at −80 °C under ambient pressure. These were similar conditions, as reported previously by our group [18,19,21,22,24,25,26].

#### 2.2.2. Scanning Electron Microscopy (SEM)

Using similar conditions as previously reported [18,19,21,24,25,26,27], scanning electron microscopy (SEM) of the Lactomorphin and Trehalose (as supplied by the manufacturer), SD, and co-SD powders were evaluated. Visual imaging, analysis of particle size, morphology, and surface morphology were achieved by (SEM). Powder samples were attached to aluminum SEM stubs (Ted-Pella, Inc., Redding, CA, USA) using double-sided carbon conductive adhesive Pelco tabs^™^ (TedPella, Inc., Redding, CA, USA). Subsequently, the powder sample in the stub was sputter-coated with a 7 nm thin film of gold using Anatech Hummer 6.2 (Union City, CA, USA) system at 20 μA for 90 s under an argon plasma. SEM images of the powder sample were collected using a FEI Inspect S (FEI, Hillsboro, OR, USA). Images were collected using a tungsten source at 30 kV with a working distance of 10–10.4 mm. SEM images were captured at several different magnification levels.

#### 2.2.3. Particle Sizing and Size Distribution

Using SEM micrographs, the mean size, standard deviation, and size range of the particles were determined digitally using SigmaScan Pro5.0.0 (Systat, San Jose, CA, USA). As previously reported [18,19,21,22,24,25,27]. Representative micrographs for each particle sample at 8000× magnification were analyzed by measuring the diameter of at least 100 particles per powder.

#### 2.2.4. X-ray Powder Diffraction (XRPD)

The crystalline nature (the presence of long-range molecular order) versus non-crystallinity (lack of long-range molecular order) of Lactomorphin and Trehalose (all as supplied by the manufacturer) SD and co-SD powders were examined using XRPD. The patterns of samples were collected at room temperature. The experiments were performed on a Philips PANalytical X’Pert PRO MPD instrument equipped with the copper X-ray source (Kα radiation with λ = 1.5406 Å). A high-sensitivity X’celerator X-ray detector between 5.0° and 70.0° (2*θ*) with a scan rate of 2.00° per minute at ambient temperature to reliably detect the XRD powder patterns from small amounts of powder samples the powder samples were loaded on zero background single crystal silicon holder. These were similar conditions to those previously reported [18,19,24,26].

#### 2.2.5. Differential Scanning Calorimetry (DSC)

Using conditions similar to previously reported [18,19,21,22,24,25,26,27]. Thermal analysis and phase transition measurements for raw Lactomorphin, raw Trehalose, SD, and co-SD formulations were studied. Thermograms obtained using the TA Q1000 differential scanning calorimeter (DSC) (TA Instruments, New Castle, DE, USA), with T-Zero^®^technology, automated computer-controlled RSC-90 cooling accessory, autosampler. A mass of the 1–3 mg sample was carefully weighed into hermetic anodized aluminum DSC pan. The T-Zero^®^ DSC pans were hermetically sealed with the T-Zero hermetic press (TA Instruments). An empty hermetically sealed aluminum pan was used as a reference. DSC measurements were performed at the heating rate of 5.00 °C/minute from 0.00 °C to 300 °C. UHP nitrogen gas was used as the purging gas at a purge rate of 50 mL/min. The glass transition temperature (T_g_) and the melting point (T_m_) were measured and calculated using TA Universal Analysis (TA Instruments). Furthermore, to ensure reproducibility, all measurements were carried out in triplicate (*n* = 3).

#### 2.2.6. Hot Stage Microscopy (HSM) under Cross-Polarizers

Solid-state phase transitions for the powders were observed under a cross-polarizing light hot stage microscope (HSM) for the presence or absence of birefringence using conditions similar to previously reported [19,22,24,25,26,27]. HSM studies used a Leica DMLP cross-polarized microscope (Wetzlar, Germany) equipped with a Mettler FP 80 central processor heating unit and Mettler FP82 hot stage (Columbus, OH, USA). Powder samples were mounted on a microscope slide with cover glass and placed into the hot stage sample chamber and heated at 5.00 °C/min from 25.0 °C to 300.0 °C. The images were digitally captured using a Nikon Coolpix 8800 digital camera (Nikon, Tokyo, Japan) under 10× optical objective and 10× digital zoom.

#### 2.2.7. Karl Fisher Titration (KFT)

Using conditions similar to previously reported [19,21,26,27], the residual water content of all powders was quantified by Karl Fischer titration (KFT) colorimetrically using a TitroLine^®^ 7500 trace titrator (SI Analytics, Mainz, Germany). Around 1–2 mg of sample was dissolved in 0.5 mL AQUA STAR anhydrous methanol. The sample solution was injected into a reaction cell filled with Hydranal^®^Coulomat AD reagent; then, a simple calculation obtained the sample’s water content. All experiments were done in triplicate (*n* = 3).

#### 2.2.8. Confocal Raman Spectroscopy (CRM)

CRM demonstrated utility in the non-invasive and non-destructive microspectroscopic analysis of DPI formulation by chemical imaging, using similar conditions previously reported [24,27]. Renishaw InVia Reflex (Gloucestershire, UK) was used to obtain Raman spectra at 785 nm excitation at 50× magnification objective on a Leica DM2700 optical microscope (Wetzlar, Germany). Raman spectral was performed for each co-SD-Lactomorphin:Trehalose powder at three different powder spots. Three accumulations were performed for each spot using 10 s of detector exposure time per accumulation with spectral scan from 50–4000 cm^−1^. Spectra were subjected to baseline correction before further analysis using Renishaw WiRE 3.4 software.

#### 2.2.9. Attenuated Total Reflectance-Fourier-Transform Infrared (ATR-FTIR)

A Nicolet IS50R FT-IR spectrometer (Waltham, MA, USA) equipped with an MCT-A detector and a Thunderdome attenuated total reflectance (ATR) (Spectra-Tech, Oak Ridge, TN, USA) accessory with a germanium window was used for all the experiments. Each spectrum was collected for 32 scans at a spectral resolution of 8 cm^−1^ over the wavenumber range of 4000–400 cm^−1^, spectral data were acquired and processed using OMNIC Spectra Software.

#### 2.2.10. In Vitro Aerosol Dispersion Performance

Aerosolization of the Lactomorphin (as supplied by the manufacturer), SD, and co-SD powders was evaluated, following United States Pharmacopeia (USP) chapter <601> specifications on aerosols specifications and using conditions similar to previously reported, [19,21,22,25,27]. The Next Generation Impactor™ (NGI™) (MSP Corporation, Shoreview, MN, USA), coupled with FDA-approved human DPI device Arcapta™ Neohaler™ (Novartis, Basel, Switzerland) at airflow rate (Q) of 60 L/min were used. The mass of powder deposited on each stage was quantified by the gravimetric method using type A/E glass fiber filters with inhalation grade capsules, which was filled with about 10 mg of powder. Three capsules were used in every single experiment, and all experiments were triplicated (*n* = 3) at ambient temperature and humidity. The Da50 aerodynamic cutoff diameter for each NGI stage was calibrated by the manufacturer and stated as: stage 1 (8.06 μm); stage 2 (4.46 μm); stage 3 (2.82 μm); stage 4 (1.66 μm); stage 5 (0.94 μm); stage 6 (0.55 μm); and stage 7 (0.34 μm). The Arcapta™ Neohaler™ (Novartis) was tightly inserted into the induction port via a mouthpiece adapter. The emitted dose (ED) was determined as the difference between the powder’s mass loaded in the capsules and the remaining mass of powder in the capsules following aerosolization. The ED (%) was calculated using Equation (1) based on the total dose (TD) used. The fine particle dose (FPD) was measured as the summation of all masses deposited powder on stages 2 to 7. The fine particle fraction (FPF%) was calculated using Equation (2) based on the total deposited dose (DD) on stages (1–7. The respirable fraction% (RF%) was then calculates using Equation (3). Besides, the mass median aerodynamic diameter (MMAD) of aerosol particles and geometric standard deviation (GSD) were calculated using a Mathematica (Wolfram Research, Inc., Champaign, IL, USA) program written by Dr. Warren Finlay.

(1) $$\mathrm{Emitted}\;\mathrm{dose}\left(\mathrm{ED}\%\right)=\frac{\mathrm{ED}}{\mathrm{TD}}\times 100\%$$

(2) $$\mathrm{Fine}\;\mathrm{particle}\;\mathrm{fraction}\left(\mathrm{FPF}\%\right)=\frac{\mathrm{FPD}}{\mathrm{ED}}\times 100$$

(3) $$\mathrm{Respirable}\;\mathrm{fraction}\left(\mathrm{RF}\%\right)=\frac{\mathrm{FPD}}{\mathrm{DD}}\times 100\%$$

#### 2.2.11. Solubility Studies

The solubility of the unionized form of raw (unprocessed) Lactomorphin was determined by the shake-flask method (SFM) using conditions similar to previously reported [28,29,30,31]. For each reported solubility result, three independent SFM experiments were carried out in parallel in the following solvents (purified water, PBS, NS, methanol, and ethanol). For each experiment, the solid sample of Lactomorphin was added using a spatula to a specific volume of each solvent in closed, light-protected glass vials while shaking the vial until a heterogeneous system of the solid sample and the solvent was achieved. Samples were visually inspected to ensure that solid Lactomorphin was still in excess in each vial. The solution containing solid excess of the Lactomorphin was then capped and allowed to agitate using the shaker for 24 h at room temperature (25 °C). The presence of precipitation was checked after several hours. If dissolution was complete, more solid Lactomorphin was added to the vial. 

The pH values of Lactomorphin ampholyte suspensions were adjusted to the isoelectric point to determine the solubilities of the zwitterionic species point and to assure the predominant presence of the unionized form of Lactomorphin. The pH of each Lactomorphin suspension in each solvent (except in methanol and ethanol, which were just measured) was adjusted by using 0.1 M NaOH/0.1 M HCl to a value of 8.5. The pH values for all vials were checked after saturation, several hours after the shaking process started, and after equilibrium was achieved. Separating saturated solution and precipitate by sedimentation was performed, which is more accurate than filtration since the filtration step is labor-intensive and tedious, it has been a known issue that drug loss can occur through membrane absorption during filtration in the SFM [32]. Aliquots of supernatant were then taken out with a micropipette from all saturated solutions and diluted with each corresponding solvent. Analytical HPLC measured the concentration of Lactomorphin in each aliquot in against freshly prepared calibration curves. As mentioned above, three independent SFMs were made for each reported result. Thus, the reported results were the mean of three measured concentrations and the standard deviation of the mean (*n* = 3, mean ± Standard deviation).

#### 2.2.12. Partitioning Studies

Lipophilicity is a critical physicochemical property that affects ADMET (absorption, distribution, metabolism, excretion, and toxicity) [33]. SFM is the most known lipophilicity measurement method, which is based on liquid-liquid extraction using the n-octanol/water system. The partition coefficient of Lactomorphin was measured at the room, and physiological temperatures via SFM using conditions similar to previously reported [33,34,35,36]. The Lactomorphin powder was dispensed into two individual glass vials containing equal volumes of 1-octanol and water to achieve a 1 mg/mL concentration. The water solution in each vial was saturated with octanol before analysis. The pH was measured and fixed to 8.5 value to assure the predominant presence of the unionized form of Lactomorphin. The pH stability in both vials was regularly checked throughout the experiment. The first vial was shaken for 24 h at room temperature. The other one was shaken for 24 h while kept in an incubator at 37 °C. After 24 h, the vials shaking was stopped. The vials kept in a vertical position to equilibrate at these specific temperatures without mixing for 24 h, to ensure full separation between the 1-octanol and aqueous layers. A sample from each layer was carefully withdrawn and analyzed by the analytical HPLC to measure the concentration of Lactomorphin in each layer then calculate the Partition coefficient (P) and logP using the following equations:

(4) $$P\;=\frac{\left[\mathrm{neutral}\;\mathrm{solute}\right]\mathrm{oct}}{\left[\mathrm{neutral}\;\mathrm{solute}\right]\mathrm{Aq}}$$

(5) $$\mathrm{Log}P=\log10\frac{\left[\mathrm{neutral}\;\mathrm{solute}\right]\mathrm{oct}}{\left[\mathrm{neutral}\;\mathrm{solute}\right]\mathrm{Aq}}$$

#### 2.2.13. HPLC Analysis for Solubility and LogP Experiments 

The HPLC system was LC-2010HT next generation HPLC (SHIMADZU Tokyo, Japan), comprised of a degassing unit, low-pressure gradient unit, pump unit, mixer, ultra-fast autosampler, column oven, and a UV-VIS detector with a thermostatted flow cell. System. The analysis was performed by a reverse phase HPLC assay, using Phenomenex Prodigy 5 um ODS (3) 250 × 4.6 mm HPLC Column. Ultraviolet detection was done at 280 nm. Mobile phase conditions were gradient of acetonitrile (CH_3_CN) in water (H_2_O) with 0.1% trifluoroacetic acid (F_3_CCOOH) at a 1 mL/min flow rate. The injection volume was 20 μL; the retention time for Lactomorphin was ~8.5 min. Quantification was determined using peak area and calculated from a nine-point standard curve; standard curve points were prepared by dilution in water.

#### 2.2.14. Thermal Degradation Study of Lactomorphin

Forced degradation is a degradation of new drug substances at conditions more severe than accelerated conditions. The FDA and ICH guidance estate the requirement of stability testing data to understand how the quality of drug changes with time under various environmental conditions [37,38,39,40,41,42,43]. Thermal, hydrolysis, oxidation, and photodegradation are commonly used stress study conditions. Knowledge of a molecule’s stability helps select proper formulation and provide adequate storage conditions and shelf life, which is essential for regulatory documentation [37,38,39,40,41,42,43]. As the temperature is one of the most vital factors that affect the stability of a drug material more than the other conditions [42], in this study, we performed thermal degradation by exposing samples of solid-state Lactomorphin to dry and wet heat.

Samples preparation were done by weighing 25 mg of Lactomorphin, transfer it into a 25 mL volumetric flask, and add 25 mL of water and shake so all the quantity dissolved. Using a calibrated micropipette, transfer 0.20 mL of this 1 mg/mL Lactomorphin stock solution into around 90 (2 mL) glass vials. Freeze-dry all vials so that all vials have accurately 0.2 mg of Lactomorphin after lyophilization. A saturated salt solution of NaCl to achieve about 75% Relative Humidity (RH) and saturated salt solution of P_2_O_5_ to reach about 0.00% RH were prepared. Thirty vials contain 0.2 mg of Lactomorphin were kept inside a separate desiccator (tightly closed 20 mL glass vial with saturated NaCl). Another set of 30 vials were kept inside the desiccator (tightly closed 20 mL glass vial with saturated P_2_O_5_). All were put in oven fixed at 62 °C temperature. To achieve equal distribution of heat inside the oven for all samples used for the degradation study, they were placed in a metallic block made, especially for this study. The rest of the vials were kept in a desiccator at −80 °C to be used as comparison(control) samples. 

According to the regulatory guidelines, there is no specific concentration used for the degradation study; however, 1 mg/mL concentration is recommended [37]. This study used three concentrations by dissolving the stability samples in 0.2 mL, 0.4 mL, and 2 mL water to achieve 1000 μg/mL, 500 μg/mL, and 100 μg/mL concentration, respectively. Analysis of the stability samples was performed every time against a freshly prepared calibration curve and comparison samples (control samples that freshly prepared and injected directly without being exposed to the force degradation conditions) at the following periods: 0.00, 0.08, 0.17, 1, 2, 4, 10, 20, 45, 94 and 133 days. All experiments at each time point and different stability conditions were done in triplicate (*n* = 3). The HPLC system consisted of a VARIAN ProStar model 330 PDA Detector coupled with a VARIAN ProStar Solvent delivery module and VARIAN ProStar model 430 auto-sampler. The analysis was performed by a reverse phase HPLC assay, using Inspire™ 5 μm C18, 250 × 4.6 mm HPLC Column. Ultraviolet detection was done at 280, 254 and 220 nm, but the quantitation was done at 280 nm only. Mobile phase conditions were gradient according to the above program in Section 2.2.13 and at a flow rate of 1.00 mL/min.

#### 2.2.15. In Vitro Cell Culture Assays and Cell Viability Experiments after Treated with Lactomorphin 

In vitro cell viability assays were performed to measure the toxicity of Lactomorphin in nasal epithelium cell, blood-brain barrier endothelial cells, NHA, H441, and A549 lung cells using resazurin as a marker. Human nasal epithelial cells RPMI 2650 were used between passages 9 and 13 for the experiments. Cell lines were grown in a growth medium including Eagle’s Minimum Essential Medium (EMEM) ATCC^®^ 30-2003™ (EMEM) 10% (*v/v*) fetal bovine serum (FBS), 1% (*v/v*) Pen-Strep (100 Unite/mL penicillin, 100 μg/mL streptomycin), 0.2% (*v/v*) fungizone (0.5 μg/mL amphotericin) in a humidified incubator at 37 °C and 5% CO_2_. For passage, the cells were detached when confluence reached about 80% by treating the cells with trypsin-EDTA at 37 °C. The cells were collected, and viability was determined using a standard resazurin assay for cell viability procedure. The Immortalized Human Cerebral Microvascular Endothelial (hCMEC/D3) were used between passages 29 and 31. hCMEC/D3 cells were seeded on collagen-coated culture flasks, all culture wares were coated with rat-tail collagen type I in PBS solution according to manufacture protocol [44] and as in [45,46] were incubated for at least 1 h at 37 °C and 5% CO_2_. Cell lines were grown in a growth medium, including EndoGRO™-MV Complete Media Kit (Cat. No. SCME004) supplemented with 1 ng/mL FGF-2 (Cat. No.GF003) in a humidified incubator at 37 °C and 5% CO_2_. Cell culturing and sub-culturing procedures were performed according to the manufacture protocol [44]. The medium was changed every two days until the cells reached confluence. The monolayer integrity of hCMEC/D3 cells at confluence was confirmed by expressing endothelial cell-specific phenotypic markers cell-cell junctions [47].

Normal Human Astrocytes (NHA) were used between passages 1 and 3 for the experiments. Cell lines were grown in a growth medium, including Astrocyte basal medium ABM™ (Lonza, CC-3187). The supplements for a complete growth medium AGM™ SingleQuots™ (Lonza, CC-4123) (aseptically, each supplement vial was opened and added the entire amount to the 475 mL of the basal medium) [48]. Cell culturing and sub-culturing procedures were performed according to the manufacture protocol [48]. The medium was changed every two days until the cells became 70–80% confluent and contained many mitotic figures throughout the flask. H441 lung adenocarcinoma cells (ATCC^®^ HTB174™), and A549 adenocarcinoma human alveolar basal epithelial cells (ATCC^®^ CCL-185™) were used between passages 6 and 9. Cell lines were grown in a growth medium including RPMI Medium 1640(1X) (gibco, 11875-093, 500 mL), 10% (*v/v*) fetal bovine serum (FBS), 1% (*v/v*) Pen-Strep (100 Unite/mL penicillin, 100 μg/mL streptomycin), 0.2% (*v/v*) fungizone (0.5 μg/mL amphotericin) in a humidified incubator at 37 °C and 5% CO_2_.

For passage, the cells were detached when confluence reached ~80% by treating the cells with trypsin-EDTA at 37 °C. The cells were seeded in a 96 well-plate at specific densities then exposed to different concentrations of the Lactomorphin solutions that were prepared by dissolving the Lactomorphin particles in 100% un-supplemental media. 100 μL of the Lactomorphin solution or control solution (100% supplemental media) was added to each well. Forty-eight hours after exposure, the media containing Lactomorphin was removed from each well and replaced by100 μL of media containing 20 μM resazurin sodium salt dissolved in unsupplemental media and incubated for 4 h at 37 °C and 5% CO_2_. At this point, the fluorescence intensity of the resorufin (metabolite) produced by viable cells was detected at 544 nm (excitation) and 590 nm (emission) using the Synergy H1 Multi-Mode Reader (BioTek Instruments, Inc., Winooski, VT, USA). The relative viability of the cell line was calculated as follow by Equation (6):

(6) $$\mathrm{Relative}\;\mathrm{viability}\;\left(\%\right)=\frac{\mathrm{Sample}\;\mathrm{fluorescence}\;\mathrm{intensity}}{\mathrm{Control}\;\mathrm{fluorescence}\;\mathrm{intensity}}\;\times 100\%$$

#### 2.2.16. Computational Predictions of Physicochemical Parameters (PCP) of Lactomorphin

Physicochemical parameters are the central focus in designing new formulations for newly discovered drug candidates. Lactomorphin glycosylated peptide chemical structure was used for building a molecular database (MDB) using the Molecular Environmental Operations program (MOE) (Molecular Operating Environment (MOE), 2019.01; Chemical Computing Group ULC, 1010 Sherbrooke St. West, Suite #910, Montreal, QC, Canada, H3A 2R7, 2019). Using the MOE database building technique, Lactomorphine. MDB was constructed to include the chemical structures of the parent compound and close analogs to be used as references in the validation of the calculations of descriptors of interest. The main focused descriptors were the ones related to pH titration, protomers population, solubility (h_LogS), and lipophilicity (h_LogP). Reference compounds, including parent peptide of Lactomorphin (Tyr^1^-Gly^2^-Thr^3^-Phe^4^-Leu^5^-Ser^6^-NH_2_), Lue-Enkephalin, and Met-Enkephalin, were incorporated in the database, and their corresponding parameters were computed.

For pH titration, we used Lactomorphinmmorphin-MOE.MDB, which has the chemical structure of Lactomorphin build with MOE using the extended non-natural amino acid version Ser(O-Lactomorphinse)-NH_2_ (Slac) database available in Protein Builder. The amino acid sequence was checked by using the footer option for (Atoms), and the helicity was checked using the footer for Ribbon, then we used MDB mode (MOE → Compute → Prepare → Protomer →) to generates the chemical structures of various protomers based on the selected pH. Protomer’s chemical structures were examined by sending them to the MOE window, to populate the database with other descriptors, the following command line was used (MOE → Compute → Descriptors → Calculate →) and select the targeted descriptors.

## 3. Results

### 3.1. Scanning Electron Microscopy (SEM)

**Figure 2:**
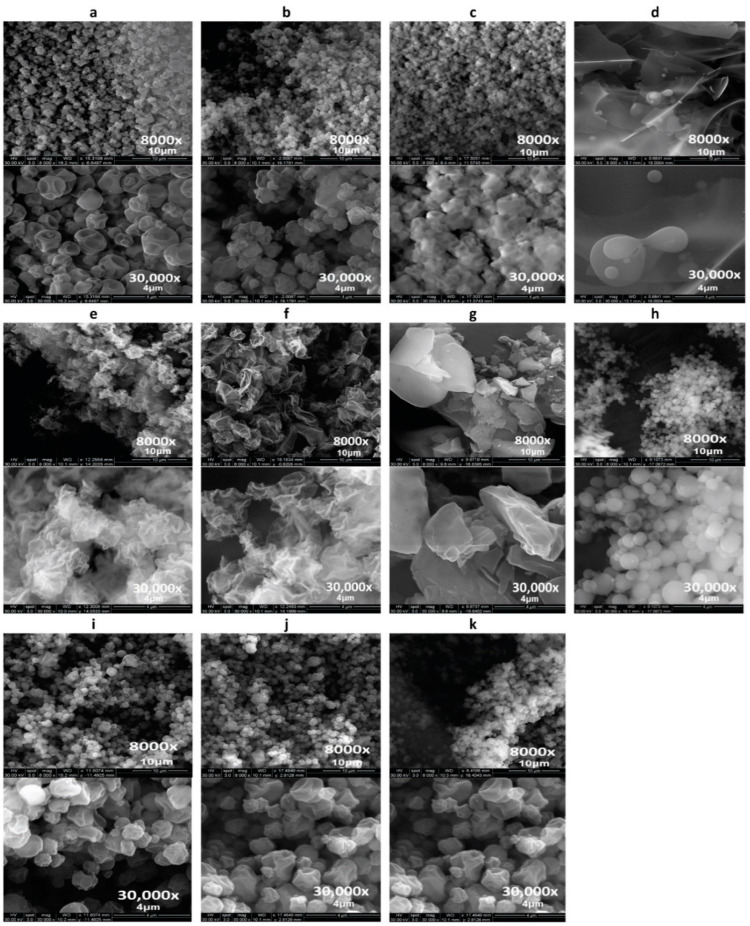
SEM micrographs at two magnifications (8000× and 30,000×) for each sample: (**a**). Raw Lactomorphin as supplied by the manufacturer; (**b**). SD-Lactomorphin in MeOH at 25% PR; (**c**). SD-Lactomorphin in MeOH at 50% PR; (**d**). SD-Lactomorphin in MeOH at 75% PR; (**e**). SD-Lactomorphin in EtOH at 25% PR, (**f**). SD-Lactomorphin in Isop at 25% PR; (**g**). Raw Trehalose as supplied by the manufacturer; (**h**). SD-Trehalose in MeOH at 25% PR; (**i**). Co-SD-Lactomorphin:Trehalose 25:75 in MeOH at 25% PR; (**j**). Co-SD-Lactomorphin:Trehalose 50:50 in MeOH at 25% PR; and (**k**). Co-SD-Lactomorphin:Trehalose 75:25 in MeOH at 25% PR.

The surface morphology of the raw non-spray-dried Lactomorphin and Trehalose (as supplied by the manufacturer), SD, and co-SD-Lactomorphin:Trehalose formulations were investigated by SEM (Figure 2). The particle size diameter, standard deviation, and range are tabulated in (Table 2), as quantified statistically using SigmaScanTM software. In this experiment, the SD pump rates and the feed solvent in the one-component Lactomorphin system were varied, then the molar ration of Lactomorphin and Trehalose was also varied to determine this effect on the particle systems. Spray-dried Lactomorphin was successfully produced at low (25%), medium (50%), and high (75%) pump rate while was failed to be produced at a high (100%) pump rate. SEM visualized the particle shape and surface morphology for all raw and spray-dried (SD) one-component powders and two-component powders of Lactomorphin and Trehalose (Figure 2). Smaller-spherical particles of SD-Lactomorphin at low pump rate (25%) in methanol (Figure 2b) compared with shriveled raw Lactomorphin (as supplied by the manufacturer) (Figure 2a), particles sintering of SD-Lactomorphin particles at low pump rate (25%) in ethanol (Figure 2e) and isopropanol (Figure 2f) and medium PR (50%) in methanol (Figure 2c), the formation of chunks-like powder of Lactomorphin at high PR (75%) in methanol (Figure 2d). 

For raw Trehalose (as supplied by the manufacturer) (Figure 2g), SEM shows irregular morphology, while the SD-Trehalose at a low pump rate (25%) in methanol (Figure 2h) shows smooth small-spherical particles. A combination of the two spray dryer’s parameters: low pump rate (25%), and methanol as the feed solvent, produced particles with a smooth smaller particle size compared with medium and high pump rates and also compared with low pump rate combined either ethanol or isopropanol separately as feed solvents. We decided to use these two parameters in the Co-SD of the two components Lactomorphin:Trehalose systems. On the other hand, increasing the Trehalose content in the co-SD powders had changed the particle morphology, as visualized by SEM, from slightly corrugated surface morphology to smoother surface morphology. Generally, all particles in the co-SD two-component system had spherical particle morphology.

### 3.2. Particle Sizing and Size Distribution by Image Analysis of SEM Micrographs 

For particle sizing and size distribution by image analysis of SEM micrographs, a representative micrograph for each sample (Figure 2 top images) at 8000× magnification was analyzed by measuring the diameter of at least 100 particles per sample as shown in (Table 2) using sigmasacn software. SD-Lactomorphin in methanol at 25% PR had a mean geometric diameter 0.80 ± 0.25 μm in size range of 0.21–1.60 μm, while the raw Lactomorphin sample had a mean geometric diameter 1.51 ± 0.71 μm in size range of 0.07–3.54 μm. The SD-Lactomorphin in methanol at 50% PR was not analyzed due to its agglomerated state. Large chunks of powder were formed for the SD-Lactomorphin in methanol at 75% PR. Varying Trehalose content from 25% to 75% in the co-SD-Lactomorphin:Trehalose samples did not significantly affect the particle size (Table 2), but the size distribution was narrower in the co-SD-Lactomorphin:Trehalose 75:25. When compare the all produced powders in all experiments, the SD-Lactomorphin at 25% PR in methanol shows the smallest particle size with the narrowest size distribution. However, all SD and co-SD batches’ particle dimensions appear smaller than that of unprocessed raw Lactomorphin.

### 3.3. X-ray Powder Diffraction (XRPD)

**Figure 3:**
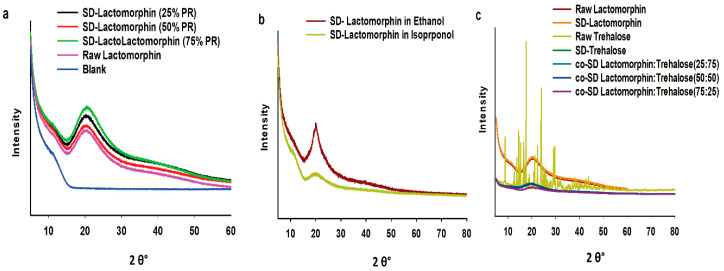
XRPD diffraction patterns for: (**a**). raw Lactomorphin, SD-Lactomorphin at all pump rates in methanol powders; (**b**). SD-Lactomorphin from two different feed solvents designed at low spray drying pump rate (25%); (**c**). raw Lactomorphin, SD-Lactomorphin, raw Trehalose, SD-Trehalose, and Co-SD-Lactomorphin:Trehalose at different molar ratios designed at low spray drying pump rate (25%).

XRPD is a direct method for determining the crystalline material structure; the crystallinity of the raw Lactomorphin, raw Trehalose, all SD, and co-SD formulations were examined by studying its XRPD. The XRPD diffractogram patterns of raw Lactomorphin, SD-Lactomorphin in methanol at all pump rates (Figure 3a), and in different feed solvent designed at low spray drying pump rate (25%) (Figure 3b) particles were without any characteristic crystalline peaks. It indicated a lack of long-range molecular order, consistent with the amorphous character. In contrast, the raw Trehalose XRPD diffractogram pattern showed multiple sharp peaks, which are characteristic of the long-range molecular order present in crystalline materials. Following organic solution spray drying, Trehalose characteristic peaks were no longer present, and this is good agreement with results previously reported by our group [49]. Thus, an amorphous state for SD-Trehalose and all co-SD Lactomorphin:Trehalose compositions, as shown in Figure 3c.

### 3.4. Differential Scanning Calorimetry (DSC)

**Figure 4:**
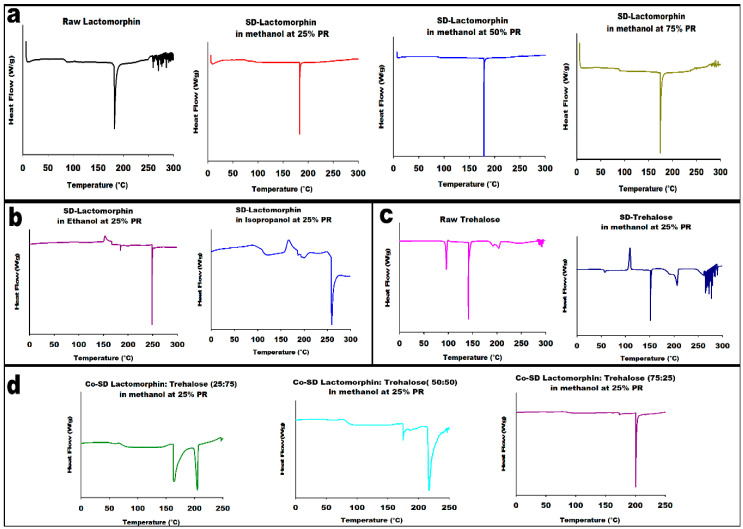
Differential scanning calorimetry thermograms of (**a**). SD-Lactomorphin particles at three pump rates versus raw Lactomorphin; (**b**). SD-Lactomorphin particles in ethanol and isopropanol at 25% pump rate; (**c**). SD-Trehalose particles at 25% pump rate in methanol versus raw Trehalose; (**d**). formulated co-SD-Lactomorphin:Trehalose at a different molar ratio in methanol at 25% pimp rate.

Thermal analysis of raw components, SD single components, and co-SD particles was done at three heating rates (5.00 °C/min (low), 20.00 °C/min (medium) and 40.00 °C/min (high)), representative DSC thermograms at 5.00 °C/min are shown in Figure 4. Thermal analysis of all DSC thermograms are summarized in (Table 3). For raw and SD-Lactomorphin in methanol at different pump rates, it was clear the components exist in an amorphous before and after spray drying; the thermograms exhibited a glass transition peak (T_g_) at a range of 82 to 92 °C. The T_g_ is a second-order solid-state phase transition from the amorphous glass to the amorphous rubber. The glass transition was followed by an endotherm peak between 170 °C and 183 °C for the raw and SD particles. Interestingly, there was a spray drying pump rate effect observed on the DSC thermograms of SD particles, an increase in T_g_, melting point (T_m_), and enthalpy was observed for SD-Lactomorphin samples with the decrease in pump rate (Figure 4a).

The thermogram of SD-Lactomorphin in ethanol and isopropanol at 25% PR (Figure 4b) shows two endothermic peaks; the first peak was a small one at ~182.00 °C and 192.00 °C, the second peak was at ~247.00 and 243.00 °C, respectively. Powders showed an exothermic peak (i.e., a disorder-to-order phase transition) suggestive of crystallization point at ~151.00 and 166.00 °C, respectively. There was no clear glass transition observed for SD-Lactomorphin in ethanol, while a clear T_g_ ~ 100.00 °C was observed for SD-Lactomorphin in isopropanol. These were in good agreement with the XRPD data, which indicated that the SD-Lactomorphin powders produced from these two alcohols were amorphous.

The thermograms for raw Trehalose and SD-Trehalose are shown in Figure 4. Three endothermic peaks were observed for raw Trehalose which were characteristic of order-to-disorder phase transitions and were in good agreement with our previous report [49]. SD-Trehalose powders from methanol at 25%PR showed an amorphous glassy-to-rubbery phase transition, T_g_, at ~53.00 °C followed by an exothermic change at ~110.00 °C, which suggests crystallization at T_c_ from the amorphous rubbery state. A small endothermic order-to-disorder peak was observed at ~145.00 °C. Similar DSC thermograms were reported by our group previously [49].

Representative thermograms from hermetically sealed DSC pans of co-SD-Lactomorphin:Trehalose in methanol at 25% PR (Figure 4d) exhibited three observable phase transitions. The glass transition, representing the solid-state transition from the amorphous glassy state to rubbery state, appeared at ~73.00 °C–82.00 °C for co-SD-Lactomorphin:Trehalose powders at different molar ratios; interestingly, the T_g_ increased as the Lactomorphin content increased from 25% to 75%; in other words, Trehalose’s presence in the co-SD system appeared not to raise the binary T_g_ values co-SD system. However, the T_g_ for the three co-SD powders were significantly above room temperature. Hence, the powders were in the amorphous glassy state at room temperature, which is consistent with the XRPD patterns. The T_g_ values are tabulated in (Table 3). An endotherm peak (i.e., order-to-disorder phase transition) at ~173.00 °C–176.00 °C was observed for co-SD-Lactomorphin:Trehalose formulations; this peak represented the T_m_ of the Lactomorphin. The third phase transition is an endothermic peak in the range of 210–215 °C, and this may relate to the T_m_ of the SD-Trehalose, the enthalpy of this transition at different molar ratio is nearly the same. There was reproducible data observed for all DSC data at higher heating scan rates of 20.00 °C/min and 40.00 °C/min, which agreed with the low heating scan rate of 5.00 °C/min for all the samples (data not shown). All DSC data agree with the XRPD data, and the degradation for all occurred at a temperature higher than 250.00 °C.

### 3.5. HSM under Cross-Polarizer Lens

**Figure 5:**
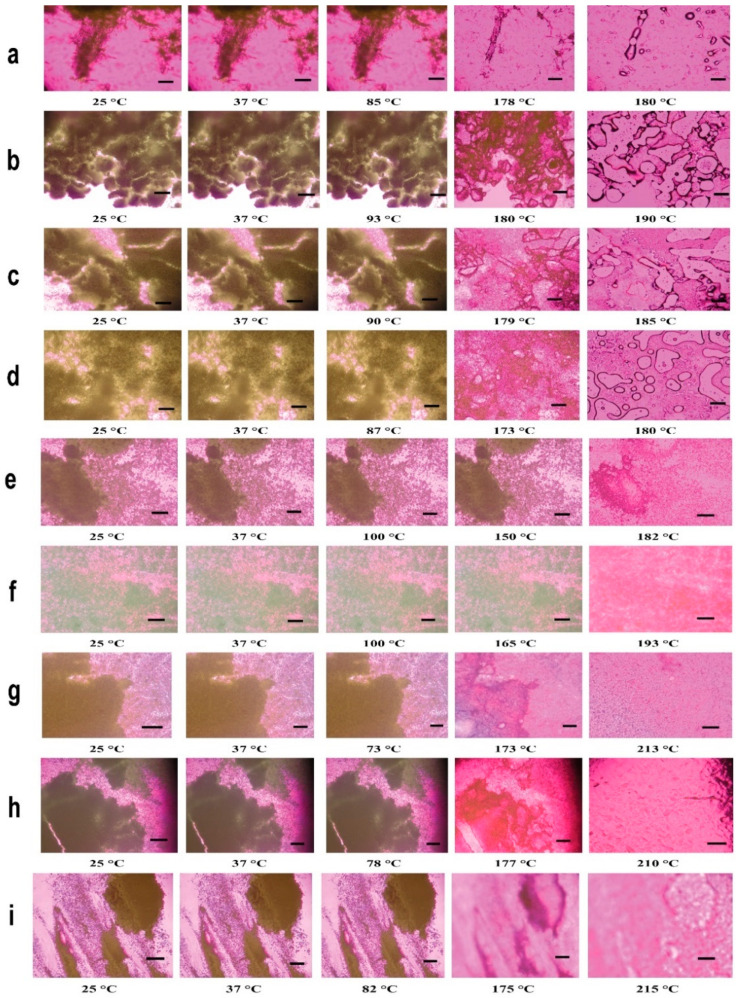
Representative HSM images for: (**a**). Raw Lactomorphin; (**b**). SD-Lactomorphin in methanol at 25% PR; (**c**). SD-Lactomorphin in methanol at 50% PR; (**d**). SD-Lactomorphin in methanol at 75%PR; (**e**). SD-Lactomorphin in ethanol at 25% PR; (**f**). SD-Lactomorphin in Isopropanol at 25% PR; (**g**). Co-SD-Lactomorphin:Trehalose 25:75 in methanol at 25% PR; (**h**). Co-SD-Lactomorphin:Trehalose 50:50 in methanol at 25% PR; (**i**). Co-SD-Lactomorphin:Trehalose 75:25 in methanol at 25% PR. Scale bar: 10 μm.

Raw Lactomorphin, SD-Lactomorphin, and co-SD-Lactomorphin:Trehalose samples at different conditions (Figure 5) showed dark agglomerates lacking birefringence over the whole temperature range under cross-polarized light which is characteristic of the noncrystalline state (amorphous state). One observable thermal event of melting (i.e., an order-to-disorder phase transition) from the solid-state to the liquid state for raw Lactomorphin and SD-Lactomorphin began to liquefy by melting at 175.0–192.0 °C (melting point). The crystallization of SD-Lactomorphin in ethanol and Isopropanol, as seen in DSC thermograms (Figure 4b) with increasing temperature, was not visually evident. That may be due to the small particle size of these two formulations (Table 2). For co-SD-Lactomorphin:Trehalose samples, the endothermic peak in the range of 210.0–215.0 °C, which may relate to the T_m_ of the SD-Trehalose, was continuing of the first endotherm transition around 175.0 °C. HSM was performed for raw and SD-Trehalose (images are not shown here); however, all the results for both powders were in good agreement with previously reported by our group [49]. The thermal behavior is complex; however, results were in good agreement with the DSC thermograms for all 11 powders visualized by HSM. The decomposition for all samples occurred at high temperatures (at least above 250 °C).

### 3.6. Karl Fisher Titration (KFT)

The water content values for all powders were analytically quantified by KF, listed in (Table 4); the water content in raw non-spray-dried Lactomorphin and Trehalose was 6.07 ± 0.67% (*w/w*) and 8.11 ± 1.96% (*w/w*), respectively. The water content values for SD-Lactomorphin in methanol at 25%, 50%, and 75% pump feed rates powders were 3.38 ± 0.23%, 3.96 ± 0.26% (*w/w*), and 4.63 ± 0.70% (*w/w*), respectively. These values for all SD-Lactomorphin powders were lower than for raw Lactomorphin powder indicating that water was removed following organic solution advanced spray drying in closed mode from a methanol solution. For these pure SD-Lactomorphin systems, the residual water content increased as the feed rate increased due to pumping more fluid per time, which lowered the energy given by the air pressure, and the temperature per droplet led to insufficient drying and higher water residue. However, all water content values remained relatively low, significantly essential for chemical and formulation stability. The mean total water content of SD-Lactomorphin at 25%PR in methanol, ethanol, and isopropanol (i.e., different feed solvents) was 3.38 ± 0.23% (*w/w*), 9.67 ± 0.71% (*w/w*), and 4.22 ± 0.15% (*w/w*), respectively. Interestingly, the water content was reduced using methanol and isopropanol but not in ethanol. The mean total water content of non-SD-Trehalose was 8.11 ± 1.96% (*w/w*), this data is in excellent agreement with the literature [49,50], while the water content value for SD-Trehalose, co-SD-Lactomorphin:Trehalose 25:75, 50:50, and 75:25 in methanol at 25% PR were 4.76 ± 0.54% (*w/w*), 4.35 ± 0.56% (*w/w*), 5.04 ± 1.05% (*w/w*) and 5.59 ± 0.38% (*w/w*), respectively. All these co-SD systems’ water content was reduced by spray drying from organic solvent (methanol) at 25% PR in closed mode relative to their respective raw powders. Furthermore, the residual water content of all co_SD Lactomorphin:Trehalose at all molar ratio’s values were close, so no effect of percentage content as a factor on the water content% after spray drying.

### 3.7. ATR-FTIR Spectroscopy

Infrared spectroscopy is one of the most powerful analytical techniques to determine the presence of various functional groups involved in making up the molecule. It provides spectral data regarding any change in a compound molecule’s functional group characteristics due to pharmaceutical processes and formulation. 

**Figure 6:**
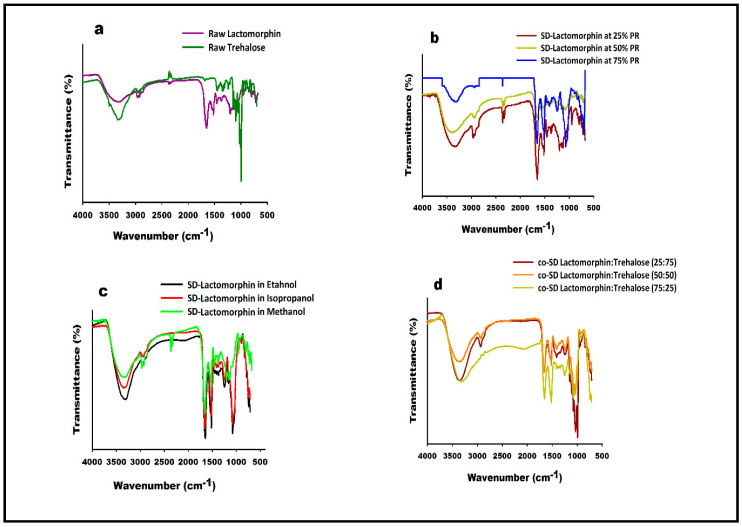
ATR-FTIR spectra of (**a**). Raw Lactomorphin and and raw Trehalose; (**b**). SD-Lactomorphin samples in methanol at different PR; (**c**). SD-Lactomorphin samples from two different feed solvents at 25% PR; (**d**). co-SD-Lactomorphin:Trehalose at different molar ratios in methanol at 25% PR.

FTIR spectra of raw Lactomorphin, raw Trehalose, and all formulations are shown (Figure 6). In the FTIR spectra of raw Lactomorphin, as supplied by the manufacturer, one prominent characteristic peak was found between 3650 cm^−1^ and 3200 cm^−1^, which was assigned to the stretching vibration of the OH group and intramolecular hydrogen bonding (Figure 6a). This band also suggested the NH stretching vibration, which was less prominent due to intense OH stretching vibration. The peak at 2900 cm^−1^ was assigned to C-H. The band at 1780–1657 cm^−1^ represented the acidic carbonyl C=O stretching. Another band at 1550 cm^−1^ to 1500 cm^−1^ represented the CH_2_ of the aromatic ring. The peak at 1374 cm^−1^ represented the hydroxyl group’s bending vibration—the band at 1250–1051 cm^−1^ assigned to C-O stretching vibrations.

These characteristic peaks also appeared in the SD-Lactomorphin at different pump rates (Figure 6b) and feed solvents (Figure 6c). Still, some peaks became more intense such as the band at 1250–1051 cm^−1^ for SD-Lactomorphin in methanol at 75% PR, SD-Lactomorphin in ethanol, and isopropanol at 25% PR. On the other hand, the band at 1780–1657 cm^−1^ and the band at 1550 cm^−1^ to 1500 cm^−1^ were less intense in SD-Lactomorphin in methanol at 50% PR with their intensity in raw Lactomorphin and the rest of SD-Lactomorphin systems.

In the FTIR spectra of raw Trehalose, a prominent characteristic peak was found between 3650 cm^−1^ and 3000 cm^−1^, which was assigned to the OH group’s stretching vibration intramolecular hydrogen bonding (Figure 6a). The peak at 2900 cm^−1^ was assigned to C-H. Another strong band at 1250–900 cm^−1^ was assigned to C-O. ATR-FTIR spectra in Figure 6d confirms the presence of the components in the co-SD particles, i.e., the presence of peaks characterize of functional groups of Lactomorphin and Trehalose in the co-SD powders at all ratios. The band at 1250–900 cm^−1^ which is a characteristic functional group of Trehalose was getting stronger as the Trehalose content increase, while the peak strengths at 1780–1657 cm^−1^ and at 1550 cm^−1^, which are characteristic functional groups of Lactomorphin were getting weaker with increasing content of Trehalose and decreasing the content of Lactomorphin. However, the presence of these characteristic functional groups peaks of Lactomorphin and Trehalose suggested that there was no incompatibility between the Lactomorphin and Trehalose molecules in all co-SD binary systems.

### 3.8. Confocal Raman Microspectroscopy (CRM)

**Figure 7:**
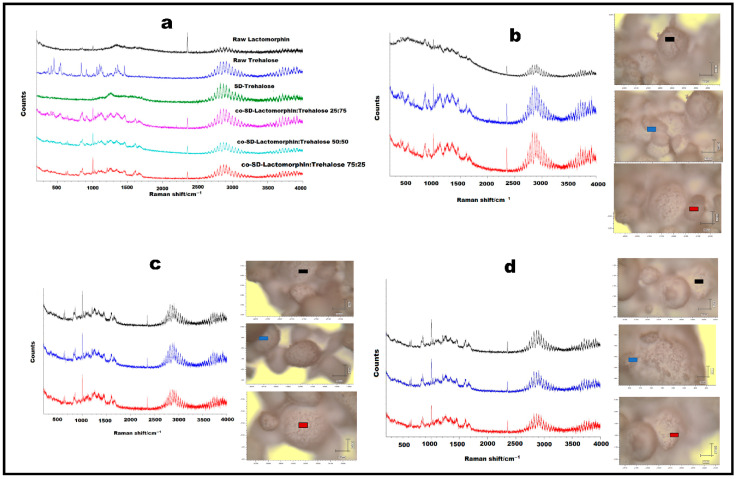
Raman spectra of (**a**). one-component powders for Raw Lactomorphin, raw Trehalose, and SD-Trehalose and two-component powders at different molar ratios; (**b**). Raman spectra for co-SD-Lactomorphin:Trehalose 25:75 at three regions with the microscopic images of the powder in each spot Scale bar: 100 μm; (**c**). Raman spectra for co-SD-Lactomorphin:Trehalose 50:50 at three regions with the microscopic images of the powder in each spot Scale bar: 100 μm; (**d**). Raman spectra for co-SD-Lactomorphin:Trehalose 75:25 at three regions with the microscopic images of the powder in each spot Scale bar: 100 μm.

Raman microscopy analysis was performed to further investigate the physical form and homogeneity of Lactomorphin and Trehalose in co-SD particles; a spectral scan from 50–4000 cm^−1^ was performed on all samples. All the samples exhibited no crystallinity before and after spray drying except the raw Trehalose, which exhibited high crystallinity before spray drying but no crystallinity after spray drying. (Figure 7a) shows the characteristic peaks corresponding to RM analysis based on the raw components’ spectral scan and their co-SD powders. Many Raman shifts were observed; the first one was in the range of 2800–3300 cm^−1^ in raw, SD and, co-SD powders. Another Raman shift in a range of 2300–2400 cm^−1^ was observed in raw Lactomorphin and all co-SD particles. Un intense Raman shifts at 1000 cm^−1^ in raw Lactomorphin, raw Trehalose, and SD-Trehalose, but it was more intense in all co-SD particles. Raman shift in a range of 1500–1600 cm^−1^ was observed in raw Trehalose and all co-SD particles, Raman shift in a range of 800–900 cm^−1^ in raw Trehalose and all co-SD particles, and low intense Raman shift in a range of 600–700 cm^−1^ in raw Trehalose and all co-SD particles.

By RM, all co-SD samples (all molar ratios in methanol and at 25% PR) exhibited homogeneity, which was probed by measuring three different locations on each sample. (Figure 7b–d) show representative brightfield micrographs obtained at 50× magnification of co-SD samples and the corresponding Raman signal obtained from different imaged samples regions. Each colored square in each image represents the powder sample’s specific spot used to assess the chemical composition. The corresponding spectra are shown (same color of the square). The peaks in the three spectra for each powder from different spots were consistently seen, suggesting a uniform distribution of the components. As shown in Figure 7a, Raman spectra were taken from the raw Lactomorphin, SD Trehalose, and co-SD Lactomorphin:Trehalose powders by RM were in general indistinguishable, suggesting that the particles were uniformly amorphous, while the raw Trehalose showed high crystallinity and these were in a good agreement with DSC, XRPD, and HSM. Figure 7b–d suggest Lactomorphin and Trehalose were homogenously mixed and did not indicate heterogeneity between the two components at the high magnification used here. Spectra were taken from three regions of each powder supported this conclusion and suggested the co-SD particles are amorphous. Additionally, the spectra showed the existence of characteristic groups of the two components, Lactomorphin and Trehalose, in the co-SD powders. There was a correlation between these two components of the binary mixture with no evidence of heterogeneity.

### 3.9. In Vitro Aerosol Dispersion Performance

**Figure 8:**
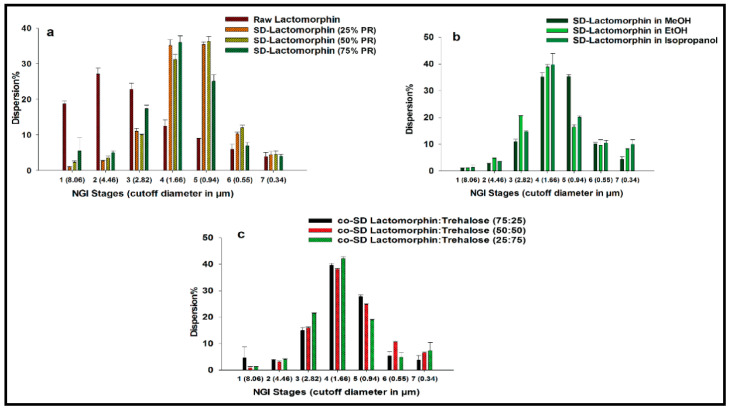
In vitro aerosol dispersion performance as DPIs using the NGI and the FDA-approved human DPI device, the Neohaler^®^ for: (**a**). Raw Lactomorphin, SD-Lactomorphin in methanol at different pump rates; (**b**). SD-Lactomorphin from different feed solvents at 25% PR; (**c**). Co-SD-Lactomorphin:Trehalose at different molar ratios in methanol at 25% PR.

Figure 8 shows in vitro aerosol dispersion performance profiles using NGI^®^ at Q = 60 L/min. As indicated in the profile, all powders performed as dry powder aerosols with measurable aerosol deposition on all NGI stages, including the lowest NGI stage, stage 7. Raw Lactomorphin exhibited high particle deposition on stages 1–3 and low particle deposition on stages 5–7. However, the spray drying process for all SD-Lactomorphin and inclusion of Trehalose in all co-SD-Lactomorphin:Trehalose systems had a profound effect on the stage deposition. As shown in Figure 8 all SD-Lactomorphin and co-spray drying Lactomorphin with Trehalose systems decreased the deposition on stages (1–3) but increased the aerosol deposition on stages 4 and 5, which include nanoparticles in the solid-state. The aerosol dispersion performance parameter values are listed in (Table 5). Raw Lactomorphin, all SD and co-SD systems had high ED values. For all SD and co-SD-Lactomorphin:Trehalose systems, the FPF was improved (45.92 ± 5.53 to 66.21 ± 5.17) compared to the raw Lactomorphin powder (27.86 ± 0.94). Also, the MMAD of less than 2.21 μm from Neohaler^®^ under Q of 60 L/min for all SD and co-SD systems compared with MMAD of raw Lactomorphin (4.29 ± 0.24). The GSD decreased, indicating the aerodynamic size distribution became narrower in the SD and co-SD systems, especially in the SD-Lactomorphin in methanol 25% PR system (1.83 ± 0.06), SD-Lactomorphin in methanol at 50% PR system (1.85 ± 0.10), and co-SD-Lactomorphin:Trehalose 75:25 in methanol at 25% PR (1.84 ± 0.17) compared with the GSD of raw Lactomorphin(4.05 ± 0.74).

### 3.10. Chromatography

The gradient HPLC method eluted the Lactomorphin at 8.4-min retention time, giving a gaussian shaped. The method was shown to be linear over the working concentration range of 7.813 to 3040 μg/mL with correlation coefficient R^2^ more than 0.99 and accuracy of calculated concentration of each calibration curve point equal 100% ± 15 as can be seen from. This HPLC method was used to analyze all solubility and LogP studies.

### 3.11. Solubility

**Figure 9:**
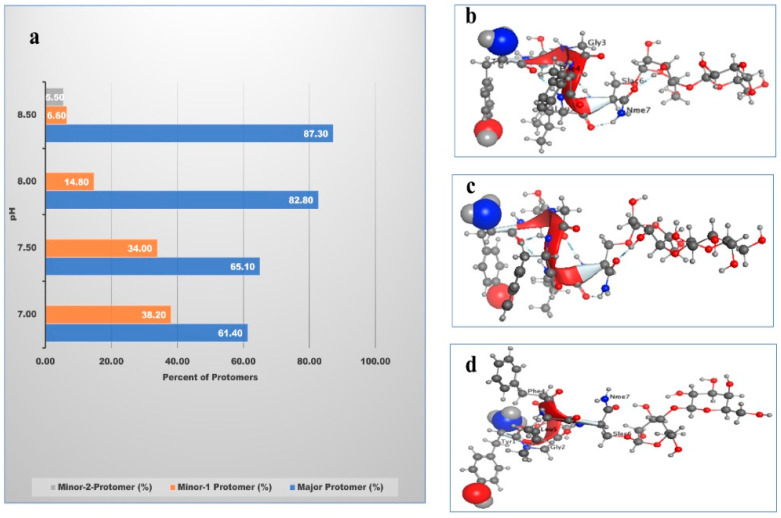
Computational pH titration of Lactomorphin (**a**). Protomers percentages at various pHs; (**b**). Major protomer 3D-structure; (**c**). Minor-1 protomer 3D-structure; (**d**). Minor-2 protomer 3D-structure.

A computational method was used in conjunction with the experimental approach to reach the isoelectric point. In the computational process, the Lactomorphin structure database was established using the MOE program’s cheminformatics approach. Lactomorphin structure was exposed to a range of pHs (Table 6 and Figure 9a) to predetermine the suitable pH in which Lactomorphin will exist in major neutral form. The results demonstrated that pH 8.5 was the right pH to maintain the neutrality of Lactomorphin with a major protomer of more than 87% with both Tyr^1^ (-α-NH_2_) and (-p-OH) groups were in their neutral ionic forms (Figure 9b). Two minor isomers were expected to be present in the solution, minor-1 present at 7% in which the Tyr^1^ phenolic -OH will be negatively charged form (Figure 9c), and minor-2 present at about 6%, and its main ionization group is the Tyr^1^-(-α-NH^3+^) group (Figure 9d). 

Using the computational approach, pH8.5 was selected to study the solubility behavior of the raw (unprocessed) Lactomorphin experimentally in various solvents. As shown in (Table 7), the experimental measurements of Lactomorphin in water, PBS, and NS are 267.00 ± 6.00 mg/mL, 570.33 ± 2.00 mg/mL, and 140.37 ± 0.32 mg/mL, respectively. According to the USP solubility definitions, all these solubility values fell in the range of 100–1000 mg/mL, which is the freely soluble category. Lactomorphin was found soluble in methanol with a 45.67 ± 1.00 mg/mL solubility and sparingly soluble in ethanol with a solubility of 18.00 ± 0.00 mg/mL. Table 7 also shows that the solubility of Lactomorphin in an aqueous environment using the experimental method (LogS of −0.60) is very close to the computational value (h_LogS of −0.65) using MOE; this result gave high confidence in our experimental determination of solubility. The computational methodology for solubility is based on the application of the Hueckel theory to build a theoretical method for the determination of the solubility of various kinds of compounds [51].

### 3.12. Partitioning Study (LogP)

Lipophilic properties were determined by using analytical instrumental techniques and computational methods. In the experimental approach, SFM was used to determine the partition coefficient (LogP) experiment of Lactomorphin. After injecting the samples to the analytical HPLC, the chromatograms (not shown here) showed a large sharp Lacomprphin peak in the aqueous layer versus a tiny peak in the organic layer. The area under the curve of each Lactomorphin peak in each layer was calculated. LogP was experimentally determined at room temperature (*n* = 3, mean ± standard deviation), and the physiological temperature (*n* = 3, mean ± standard deviation) parallel, and found to be −2.40 ± 0.00 at room temperature and −2.33 ± 0.02 at the physiological temperature as shown in (Table 8). It is worth mentioning that the pH values were adjusted from 7.2 to 8.5 to have the net charge of the Lactomorphin around zero since logP values should be measured at a pH at which the ionization of any ionizable groups is suppressed.

**Figure 10:**
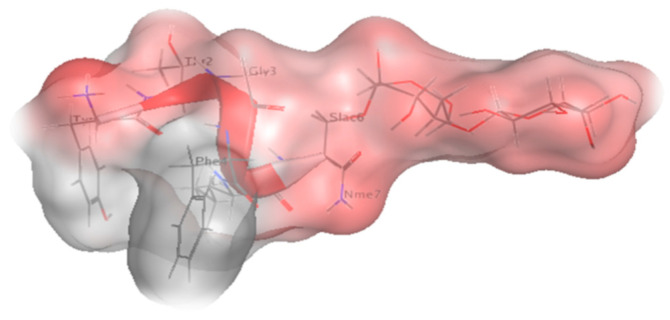
Molecular Surface of Lactomorphin with Hydrophilic (**red**) and Lipophilic (**gray**) zones.

In the computational approach, MOE was used to calculate the descriptor of h_LogP and compared it to the experimentally determined LogP, as shown in (Table 8). The computational approach allowed us to look at the results relative to the protomers’ level in the solution, the solubility of Lactomorphin in octanol, and the aqueous medium was dictated by its ionization status, which reflected in its distribution in the two layers of the immiscible solvent components. The results show comparable values between the experimental (LogP of −2.40) and the computational (h_logP of −2.76) approach. The difference of about 15% between the two methods could mainly result from the analytical method’s error versus the computational calculation. The computational approach was based on the same process used to estimate solubility using the Hueckel theory [51]. Figure 10 was built using MOE software to show the molecular surface of Lactomorphin with the hydrophilic and lipophilic zones.

### 3.13. Thermal Degradation Study

**Figure 11:**
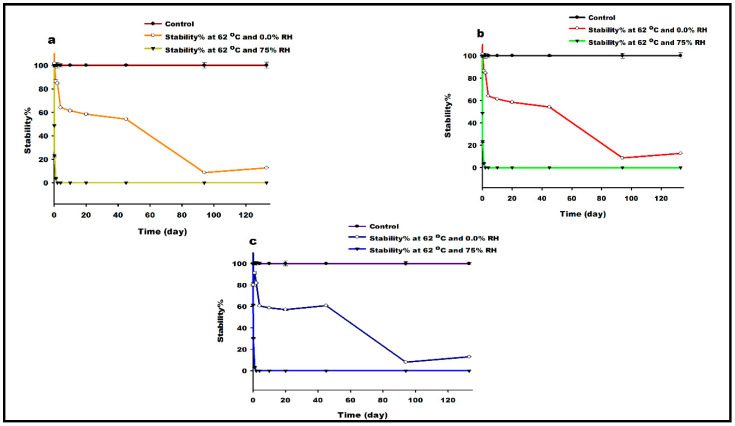
Thermal degradation profile of stored Lactomorphin powder under dry and wet heat conditions, followed by reconstitution with water to achieve concentration (**a**). 100 μg/mL; (**b**). 500 μg/mL; (**c**). 1000 μg/mL. The control samples were stored Lactomorphin powder at −80 °C in a desiccator (not exposed to heat or high humidity) reconstituted in water to achieve corresponding concentrations. Injected immediately after reconstitution. (*n* = 3, mean ± standard deviation).

The results of thermal degradation stability studies are shown in Figure 11. The percentage of Lactomorphin (remaining%) in the test-stability vials exposed thermal degradation conditions to their freshly prepared equivalent concentration controls was calculated. Stability% of around 100% was found for the control samples (*n* = 3), which were freshly prepared and directly injected, after calculating their concentrations and compare them with the theoretical ones. Lactomorphin at three different concentrations (100, 500, and 1000 μg/mL) undergoes speedy degradation after exposure to (high temperature 62 °C/high RH 75%); under these conditions, the Lactomorphin stability% dropped to 59% within 2 hrs, 29% within 4 hrs and around a complete degradation occurred within 24 h with stability% value of 6%. Interestingly, it took 10 days for Lactomorphin stability% to drop around 60% after the exposure to (62 °C/RH0.00%) conditions and stayed around this value up to day 45, a complete degradation (~10% stability) occurred on day 94, and same stability% value found on day 133.

### 3.14. In Vitro Cell Culture, Development of Cell Monolayer and Cell Viability Test 

**Figure 12:**
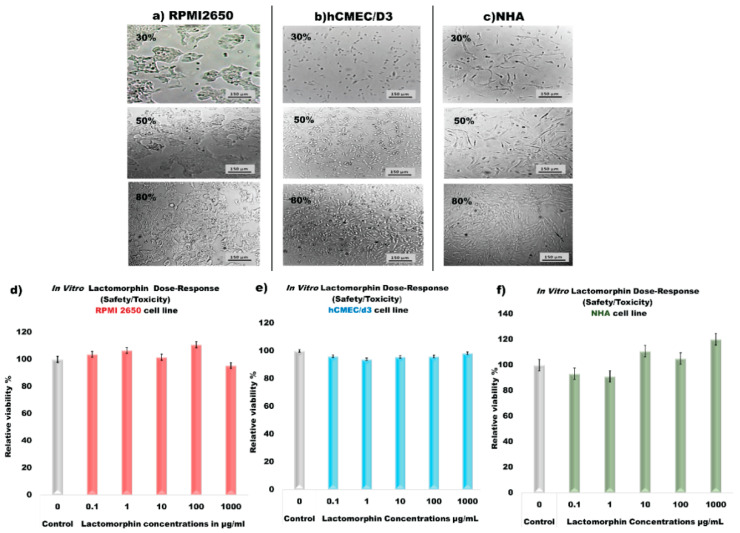
Cells morphology and at 30%, 50%, and 80% confluency during the sub-culturing as imaged with a phase-contrast microscope. Scale bar: 150 μm of (**a**). RPMI2650 cell line; (**b**). hCMEC/d3 cell line; (**c**). NHA cell line; Resazurin assay results of the cell viability experiment after the exposure to different concentrations of Lactomorphin for (**d**). RPMI2650 cell line; (**e**). hCMEC/d3 cell line; (**f**). NHA.

RPMI 2650 cell culture and development of cell monolayer RPMI 2650 cell line was successfully cultured in (EMEM) ATCC^®^ 30-2003™, with 10% (*v/v*) fetal bovine serum (FBS); 1% (*v/v*) Pen-Strep (100 Unite/mL penicillin, 100 μg/mL streptomycin); 0.2% (*v/v*) fungizone (0.5 μg/mL amphotericin) with fast proliferation and 80% of confluence after 3–4 days. By observation of monolayers under a light microscope (Figure 12a) shows 30%, 50%, and 80% confluency of RPMI2650, it was possible to verify the small size of RPMI 2650 cells and their ability to spread over the entire surface of the t-flask, reaching confluence, as reported by [52]. hCMEC/D3 cell line was successfully cultured in the growth medium, including EndoGRO™-MV Complete Media Kit (Cat. No. SCME004) supplemented with 1 ng/mL FGF-2 (Cat. No.GF003) with fast proliferation and 80% of confluence after 3–4 days. By observation of monolayer under a light microscope (Figure 12b) shows 30%, 50%, and 80% confluency of hCMEC/D3, it was possible to verify the size of hCMEC/D3 cells and their ability to spread over the entire surface of the T75 flasks coated with rat tail collagen type I in PBS. For NHA cell line was successfully cultured in a growth medium, including Astrocyte basal medium ABM™ (Lonza, CC-3187) and the supplements for a complete growth medium AGM™ SingleQuots™ (Lonza, CC-4123) with fast proliferation and 80% of confluence after 3–4 days. By observing monolayers under a light microscope (Figure 12c) shows 30%, 50%, and 80% confluency of NHA, it was possible to verify the size of NHA cells and their ability to spread over the entire surface of the T75 flasks. 

The resazurin assay for cell viability experiment shows that RPMI2650 cells (Figure 12d), hCMEC/D3 cells (Figure 12e), and NHA cells (Figure 12f) are safe after 48 h of exposure to different concentrations of Lactomorphin. There was no statistically significant difference in relative cell viability% between the control cells (not treated with Lactomorphin) and the cells exposed to Lactomorphin at concentrations of 0.1 µg/mL, 1 µg/mL,10 µg/mL, and 1000 µg/mL (*p* values > 0.05).

**Figure 13:**
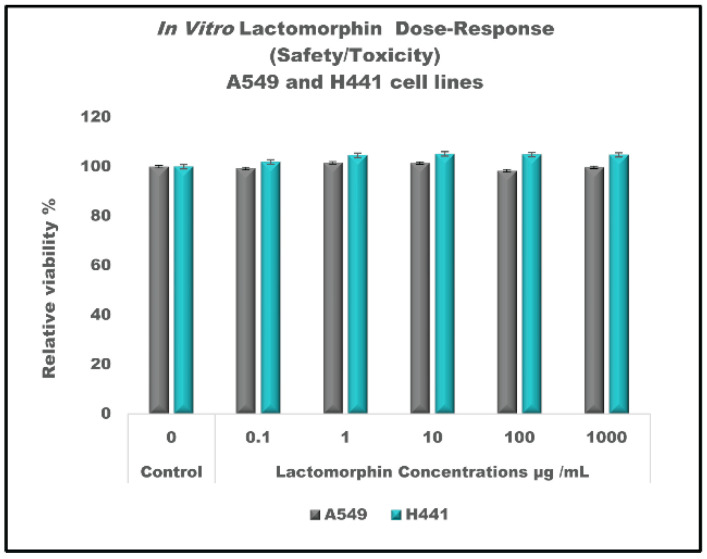
Resazurin assay results of the cell viability experiment after the exposure to different concentrations of Lactomorphin for A549 and H441 cell lines.

To check the effect of Lactomorphin on the lower respiratory tract, we chose two representative cell lines, H441 lung adenocarcinoma cells, and A549 adenocarcinoma human alveolar basal epithelial cells. Both cell lines successfully cultured and developed monolayer in in a growth medium including RPMI Medium 1640(1X) (Gibco, 11875-093, 500 mL),10% (*v/v*) fetal bovine serum (FBS), 1% (*v/v*) Pen-Strep (100 Unite/mL penicillin, 100 μg/mL streptomycin), 0.2% (*v/v*) fungizone (0.5 μg/mL amphotericin). Reaching 80% confluency within 2–3 days. The resazurin assay for cell viability experiment showed that A549 cells and H441 cells (Figure 13) are safe after 48 h of exposure to different concentrations of Lactomorphin. There was no statistically significant difference in relative cell viability% between the control cells (not treated with Lactomorphin) and the cells exposed to Lactomorphin at concentrations of 0.1 µg/mL, 1 µg/mL,10 µg/mL, and 1000 µg/mL (*p* values > 0.05).

## 4. Discussion

Noninvasive delivery strategies were extensively explored to avoid needles; among these, inhalation delivery proved to be especially attractive. The purpose of this study was to produce spray-dried (SD) powders of Lactomorphin and co-spray-dried (co-SD) powders of Lactomorphin:Trehalose with favorable physicochemical properties tailored for respiratory delivery; upper to target the brain through the olfactory route to bypass the BBB, i.e., nose-to-brain delivery, and also, this could be used as inhalations to target the lungs, i.e., the lower respiratory tract. Although the nasal cavity offers an attractive alternative approach to target the brain via the olfactory; there are many limitations of this route of administration, such as a restricted volume administration(25–200 μL) and the enzymatic activity that can degrade peptides [52], and glycopeptides such as Lactomorphin the compound of interest in this study. One of the most promising solutions is to formulate the drugs into solid-state as DPIs to increase the physical and chemical stability of the drugs [52] and facilitate the reach of the drug to the site of action fast by optimizing the formulation, including the delivery device.

Moreover, we wanted to target the lungs (lower respiratory tract); as pulmonary inhalation drug delivery offers attractive advantages in delivering high concentrations of the drug directly to the disease site in the lungs, minimizing systemic bioavailability. The human lung’s surface area is large. Its epithelium is highly permeable and easily accessed by an inhaled dose. Drug inhalation enables rapid and predictable onset of action induces fewer side effects than other administration [53]. Lactomorphin is used for pain relief, and it is a μ and δ opioid receptors agonist. The respiratory tract contains opioid receptors that are located throughout the respiratory tract; the most abundant sites within the respiratory tract appear localized within the alveolar walls. Other sites seem to line the smooth muscle within the trachea and main bronchi near the lumen [54]. Another study provides in vivo evidence for μ and δ opioid receptors in human lung carcinoma [55]. This shows the high potential of the antinociception effect of Lactomorphin if delivered to the lower respiratory tract and deep lungs.

To the authors’ knowledge, this is the first research to successfully design and optimize DPIs of Lactomorphin and (Lactomorphin:Trehalose) molecular mixtures to target the respiratory tract. Followed by comprehensively characterized the produced powders for their physicochemical properties in the solid-state. To make DPIs, we used the spray drying technique to design particles with different sizes, size distribution, and shape characteristics. This attractive technique was mainly based on a dry powder’s production following a feed solution’s atomization using a hot drying air stream [56]. Many parameters were adjusted during the spray drying process, as shown in (Table 1), including feed pump rate(low, medium, high), the solvent type (methanol, ethanol, isopropanol), the composition(pure Lactomorphin, molecular mixture of Lactomorphin and the non-reducing sugar Trehalose), and finally different molar ratios of the molecular mixture components. Regarding the other adjustable spray dryer instrument parameters such as the gas flow, aspiration%, and inlet temperature, we kept them fixed for all experiments at 670 L/h, 100%, and 150 °C, respectively. The outlet temperature was measured and noticed to be decreased as the pump rate increased with values of 31 °C, 47 °C and 76 °C at 75, 50, and 25 pump rates, respectively. Each time, one parameter was changed while keeping all other adjustable parameters fixed to study the varied parameter’s influence on the produced particles properties. In this study, we used Trehalose as an excipient in the co-CD two-component systems. Trehalose was successfully included in the inhalation formulations and played a protective role by stabilizing biostructures such as proteins and lipid membranes [57]. However, further stabilities studies to cover the stability of the co-SD two-component systems with Trehalose is needed. Our group successfully used Trehalose to produce powders suitable for pulmonary delivery as DPIs, and the physicochemical and particle properties were covered [49].

SEM images (Figure 2) and analysis (Table 2) showed interesting relationships between the feedi solvent, pump rates, the spray drying system from one side and the particle morphology, surface morphology, and the final powder’s particle size products on the other side. For the pump rate effect using methanol as a feed solvent, the low spray drying rate of 7.5 mL/min (25%) exhibited the smallest particle size, particle size distribution, and the most smooth and spherical SD-Lactomorphin particles, compared with the sintering of SD-Lactomorphin particles at 15.0 mL/min (50%) (medium pump rate) and chunk-like powder at 22.5 mL/min (75%)(high pump rate) while no powder was produced at 30.0 mL/min (100%) (high pump rate). The feed solvent type at 7.5 mL/min (25%) (low pump rate) also significantly affected particle morphology and surface morphology. Thus, methanol produced spherical particles with smoother morphology and surface morphology than ethanol and isopropanol as feed solvents. Changing the Lactomorphin:Trehalose molar ratios showed no significant effect on particle size, since the formulation composition determined the film properties and thus the particle morphology. In general, the protein or peptide drug has a stronger influence on particle morphology due to its sensitive nature to the air-water interface [58], which would influence the particle size. However, all the co-SD samples have smaller particle size than the raw Lactomorphin which could be due to the favorable intermolecular interactions between the hydroxy groups on the Trehalose molecules and the hydroxy groups on the Lactomorphin molecules. Another reason was using a low pump rate in all these co-SD systems where it was found from SEM analysis of the pure SD Lactomorphin that the pump rate played a significant role in producing particles with different particle sizes and morphology. Thus, a low pump rate for all co-SD systems led to similar particle size and morphology for the compositional ratios studied.

The olfactory epithelium, or olfactory mucosa, is located at the very top of the nasal cavity between the superior turbinate and the cribriform plate of the ethmoid bone [59]; only a tiny fraction of therapeutic agents deposit in the olfactory region and enter the brain. If drugs can be delivered deep and high enough into the nasal cavity, they will reach the olfactory mucosa and enter the brain. Therefore, it is essential to pay attention to the factors that enhance the nose-to-brain delivery and affect particle deposition in the human nose, such as particle size, airflow rate, and particle shape. The particle deposition was reported in [60] using particles with sizes range 1–100 nm that the relationship between particle size and particle diffusivity, olfactory deposition efficiency was highest for the smallest particles in both humans and rats. Among the particles that do deposit, the larger nanoparticles have a higher probability of depositing in the olfactory region. The larger nanoparticles had a more uniform deposition throughout the nasal passages [60]. Another study [61] showed that 1 nm diffusive particles, and 10 µm inertial particles, are very easily trapped by the nasal vestibule due to dominant Brownian motion and inertial force, respectively, while for particles in the range of 10 nm to 2 µm, a low olfactory deposition was observed. On the other hand, it was found that particles of 100 nm penetrated the olfactory bulb and could be found in the brain. In contrast, particles of 900 nm did not penetrate the brain, which means a particle size cutoff could be essential for the delivery beyond the olfactory bulb [62]. Although the particle size of the formulations in this study falls within a range less likely to reach the olfactory region, Lactomorphin is potent. If a small amount of this glycopeptide reaches the olfactory region, it may penetrate the brain and potentially be a successful treatment. Overall, the SEM analysis showed the SD-Lactomorphin particles produced at the low spray drying rate (25%) and methanol as a feed solvent had the best particle morphology, surface morphology, and particle size.

In this study, we used alcohol solutions to produce a smaller primary droplet size due to the lower surface tension in the range of 20–25 mN/m in contrast to water, which has a high surface tension of 72 mN/m and hence produces larger primary droplets. Table 4 shows that the SD powders had significantly lower residual water content values, as achieved by organic solution advanced spray drying under these conditions compared to raw Lactomorphin and raw Trehalose’s water content. Only SD-trehalose powder from ethanol exhibited relatively higher residual water content which was comparable to raw trehalose. For the rest of the powders, the water content values were remarkably low had a residual water content ≤ of 5.590% *w/w*. These residual water content values are considered acceptable for inhalation dry powder formulation, which is necessary for effective particle delivery since residual water can significantly influence impeding dry powder’s dispersion during aerosolization [18,21,22,23,49]. When comparing raw Lactomorphin powder vs. SD-Lactomorphin powders residual water contents, the residual water content for SD powders was reduced by spray drying from methanol in closed mode relative to the raw Lactomorphin powder. Furthermore, their relative residual water content values increased slightly with increasing pump rates; this trend is related to the fact that the pump rate impacts particle size, morphology, and water content. The feed rate refers to transferring the feed solution into the nozzle per time. It was shown that increasing the pump rate results in larger particle sizes. This can be explained by the fact that more fluid is provided; therefore, the quantity of energy given by the air pressure and the temperature per droplet is lower. This also leads to particles with higher residual water content due to insufficient drying [56].

Excellent aerosol dispersion performance (Figure 8) and optimal aerosol performance parameters (Table 5) were demonstrated using the NGI^®^ coupled with the Neohaler™ DPI device. The formulated particles would be optimal for efficient and predominant deposition into the deep lung region for targeted delivery as inhaled nanoparticles/microparticles as aerosolized powders. The MMAD values were in the optimal range of (1.64–2.21 µm) for targeting the smaller airways [23]. Furthermore, the FPF and RF values for all SD and co-SD powders were higher than the raw Lactomorphin. Raw and processed powders have nearly the same ED values in a range of (88–100%), indicating the potential for these processed systems to more efficiently deposit in the lower regions of the lung while providing high local concentration at the target site. In general, a particle’s physical properties, such as particle size, aerodynamic particle size distribution, MMAD, mass distribution, shape, and electrostatic charge, play an important role where the drug particles deposit in the lung [13]. Particle deposition in the lung occurs by inertial impaction, sedimentation, diffusion, interception, and electrostatic precipitation [63,64,65]. Impaction and sedimentation are the dominant mechanisms of deposition of therapeutic aerosols, and as lung deposition relates to particle aerodynamic behavior, the optimal MMAD for a particle is approximately 1–5 μm [13]. Other physicochemical properties that affect particle-lung interactions are van der Waals forces, hydrostatic interaction, mechanical interlocking, and electrostatic and capillary forces. They should be taken into consideration to achieve optimum MMAD value and avoid aggregation, which is very important for successful therapeutic results [13].

The absence of crystallinity in raw Lactomorphin, SD, and co-SD particles was reflected in the XRPD diffractograms (Figure 3) and confirmed by DSC thermograms (Figure 4) indicated that the novel advanced inhalable microparticles and nanoparticles exhibited a clear T_g_. This is consistent with the formation of the amorphous glassy state, which was also confirmed by visualizing the absence of birefringence through HSM (Figure 5). DSC thermal analysis (Table 3) showed a relationship between the endothermic peak values exhibited by the SD-Lactomorphin particles in methanol and the pump rates; as the pump rates increase, the melting point decreases, the same relationship between the T_g_ values and the pump rate was noticed. The glass transition temperature value depends on several factors, such as the rate of heating or cooling, the molecular weight, and the measurement method [58]. The outlet temperature(T_out_) results from the combination of many parameters such as inlet temperature, aspirator flow rate, pump rate, and the concentration of the material being sprayed [66]. T_out_ is the main factor in controlling the drying rate and essential particle characteristics, such as moisture content and particle shape [58]. All these factors were kept the same for all formulations of SD Lactomorphin except for the pump rate. As the pump rate decreases, the T outlet would increase, the particle size will decrease, the humidity would decrease, and one of the factors affecting the T_g_ is the water or moisture content; residual water can serve as a plasticizer to lower the T_g_. As the water residual% increase in the SD Lactomorphin formulations at different pump rates as quantified by KFT by increasing the PR, the T_g_ showed this proportional relationship with the pump rate; this indicates increasing the stability of the produced powder at a low pump rate. 

Interestingly, the SD-Lactomorphin particles spray-dried from ethanol and isopropanol feed solvents showed exothermic peaks below decomposition temperature while heating at 150 °C and 166 °C, respectively. In general, exothermic behavior could occur during the heating of amorphous material. The SD-Lactomorphin in isopropanol has a well-defined T_g_ indicating a significant amorphous structure that rearranges on heating to a crystalline structure before melting at about 193 °C, another endothermic peak at 243 °C appeared before the complete decomposition. The SD-Lactomorphin in ethanol has a weak T_g_, indicating an initial structure that is almost has a small crystalline percentage; this might explain the sharper peak of the XRPD diffraction pattern at 20 (2theta degree) shown in Figure 3b, another endothermic peak at 247 °C appeared before the complete decomposition. All the co-SD Lactomorphin:Trehalose powders at different molar ratios exhibited clear T_g_ values in a range of (73–82 °C), which increase as the Lactomorphin molar ratio in the two components systems increases, all the T_g_ values were higher than the physiological temperature, which enhance the stability of these powders in general. Two endothermic peaks represent the melting points of both Lactomorphin and Trehalose appeared in the DSC thermograms of the three co-SD systems with consistent and reproducible ΔH (enthalpy) values of the non- reducing sugar Trehalose as shown in (Table 3), which indicates a good encapsulation of Lactomorphin and Trehalose during the spray drying process.

ATR-FTIR analysis (Figure 6) on the co-SD particles in their dry state compared to their raw components confirmed the presence of both Lactomorphin and Trehalose, where appropriate, through each of the signature peaks of these materials without the destruction of particles that would occur using other FTIR methods. Furthermore, Raman microscopy (Figure 7) confirmed the chemical homogeneity of the particles.

In this study, the solubility and LogP of raw (unprocessed) Lactomorphin were determined experimentally and computationally, and the results from both approaches were very close (Table 7 and Table 8). The solubility and lipophilicity are from the most important physicochemical properties in the pharmaceutical research that affect the pharmacokinetics of drug release; therefore, their determination should take place in the early stages of drug research lead selection and optimization during drug discovery and development phases [30,33]. Lactomorphin is an amphipathic glycopeptide, as shown in Figure 10; it contains a carbohydrate moiety that affects the peptide and biological membranes interactions. Polt and coworkers suggested that glycopeptides have a biousian character (two essences), referring to the two different conformational states a biousian glycopeptides can adopt in the aqueous bulk compartment and when bound to the membrane. As glycopeptides have relatively lipophilic backbones and hydrophilic sugar moieties, this allows a glycopeptide to interact with both the membrane surface and aqueous compartment in a “hopping” motion, which promotes transport of the drug throughout the body in vivo and improvs the bioavailability [67,68,69,70]. Lactomorphin was found to be freely soluble in water, NS, and PBS with an aqueous solubility of >100 mg/mL. Lactomorphin was found to be soluble in methanol and sparingly soluble in ethanol. From the logP calculations, Lactomorphin was practically insoluble in 1-octanol, indicating the solubility of Lactomorphin decreases significantly as the number of carbon atoms in the organic solvents (alcohol here) increases.

The lipophilicity (logP) of Lactomorphin was quantified experimentally by measuring the extent of distribution between an aqueous phase and a hydrophobic phase 1-octanol. SFM was used rather than other experimental measures of lipophilicity such as chromatographic retention time measurement, pH metric, microfluidic liquid-liquid extractions, immobilized artificial membrane affinity, and micellar emulsion electrokinetic chromatography [34]. Hence, the concentration ratio of the neutral form of Lactomorphin in both phases was determined, LogP was calculated at room and the physiological temperatures parallel and found to be approximately the same (−2.40 ± 0.00 at room temperature and −2.33 ± 0.02 at the physiological temperature). The resulting data from the experimental and computational approaches were very close demonstrating the hydrophilicity of Lactomorphin and confirmed the solubility results. The effect of pH on the solubility of drugs with ionizable moieties is well-known, hence Lactomorphin was kept in its unionizable form by adjusting and monitoring the aqueous solvents’ pH value for the solubility and LogP experiments.

In general, forced degradation studies and thermal degradation studies are essential in pharmaceutical research and development. It is strongly recommended that these studies start as early as possible to develop stable formulations, provide information about a drug’s degradation pathways, the storage conditions, assess the inherent stability of a drug, and improve formulation and the manufacturing process [41]. One of the main aims of this study was to speed up the thermal degradation protocol of Lactomorphin since thermal degradation tests should be performed on active pharmaceutical ingredients and doses form with or without humidity [40,41,43]. Accordingly, a thermal degradation study of solid Lactomorphin was carried out at 62 °C temperature combined with low and high humidity for 4 months. The stability was assessed at three different concentrations (100, 500, and 1000 μg/mL). Lactomorphin underwent a complete degradation within 24 h with a 6% stability value after exposure to high temperature 62 °C and high RH (75%). While it took 10 days for Lactomorphin stability% to drop around to 60% under 62 °C and RH (0.00%) conditions and stayed around this value up to day 45, an approximately complete degradation (~10% stability) occurred on day 94 and same stability% value found on day 133. These data show the synergetic effect of humidity combined with high temperature on the stability of Lactomorphin. This indicates the need to store the powder under desiccators and avoid humidity exposure.

Force degradations studies aiming for a degradation level of approximately 5–20% [41], excess degradation may be misled by the interruption of the results, so it is recommended to repeat this study in less severe conditions such as lower temperature (40–50 °C) and lower humidity%. However, our target was mainly to know the suitable storage conditions of Lactomorphin, so this study was sufficient for our purpose. Regarding the degradation products, they are expected to be mainly the amino acids compose this glycopeptide and the sugar moiety. A brown color appeared in the tested-stability vials upon degradation during the experimental process, which may indicate the occurrence of the Maillard reaction between the carbonyl group of the sugar moiety and the nucleophilic amino group of the amino acid.

During the development of intranasal drug delivery systems for local or systemic effect or brain targeting, it is necessary to assess its cytotoxicity nasal epithelium to avoid animal experiments or excised tissues using in vitro cell models. As the target of this study is to deliver Lactomorphin to the respiratory tract through the intranasal route of administration as DPIs to target the brain, or as inhalation to the lung; it was important to assess the safety of Lactomorphin in cell lines represent the organs that would be exposed to Lactomorphin such as human nasal epithelium, brain human cell lines, and the respiratory cell lines to check the cytotoxic profile of the Lactomorphin. 

We rationally selected representative human cell lines with unique properties. The RPMI2650 human cell line was derived from an anaplastic squamous cell carcinoma of the human nasal septum, and resemble normal human nasal epithelium cells to karyotype [71]. Among the in vitro cell lines available commercially, RPMI 2650 is the only immortalized human nasal cell line [72] that can be differentiated over many passages. The hCMEC/D3 cell line was derived from human temporal lobe microvessels isolated from tissue excised during surgery to control epilepsy [44]. It was developed to facilitate the study of the BBB in vitro and retain critical features of primary cells, such as the expression of endothelial cell markers, transporters, and tight junctional proteins [47,73]. Astrocytes play varied roles in the healthy and diseased human brain. They have multiple housekeeping functions, including modulation of synaptic function, intracellular communication via gliotransmission and gap junctions, regulation of cerebral blood flow, and extracellular environment maintenance. They can undergo morphological and functional changes in response to disease-specific stimuli [74]. Hence, the NHA cell line was selected as one of the representative brain cell lines to check the brain toxicity after being exposed to Lactomorphin.

In the cell viability assays for the above cell lines, monolayer cultures were used as they are already recognized to be a good model for cytotoxicity evaluation of several compounds and excipients [52]. The results of toxicity experiments revealed that Lactomorphin did not show cytotoxicity on human cell lines of: nasal epithelium, endothelial, astrocyte brain cells, and lung cells in the tested concentrations range relative to the control (100% of cell viability, culture medium) after at least 48 h of incubation (Figure 12 and Figure 13). The safety of Lactomorphin in the tested cell lines was expected, as glycosylation improves biocompatibility and reduces toxicity [75,76]. Thus, the non-toxicity of Lactomorphin is expected as it is a glycopeptide.

## 5. Conclusions

To the best of our knowledge, this study is the first to report on the successful design of advanced spray-dried inhalable dry powders containing the glycopeptide, Lactomorphin, as SD-Lactomorphin (one-component) and co-SD Lactomorphin:Trehalose (two-component) with the necessary properties needed to be suitable for respiratory drug delivery as inhaled dry powder aerosols in the application of moderate to severe chronic pain treatment.

A comprehensive physicochemical characterization using different analytical techniques was performed to evaluate the final produced powders’ properties suitable for inhalation aerosol delivery. Particle engineering by advanced spray drying of Lactomorphin produced amorphous glassy inhalable powders with smaller-spherical particle size, lower residual water content, higher melting point, and glass transition temperatures, and less molecular mobility leading to decreased reactivity and hence increased physical and chemical stability. In general, the best formulation in terms of physicochemical characterization is the one-component SD-Lactomorphin from methanol at 25% PR. In vitro, predictive lung deposition modeling from the aerosol deposition patterns indicated that these particles would be expected to efficiently reach lower airways in high local concentration based on the aerodynamic properties. Lactomorphin was found to be safe (non-toxic) in vitro up to a dose concentration of 1000 μg/mL on different types of human brain neural cells and respiratory cells from different parts of the lung and nasal regions.

## Figures and Tables

**Table 1:** Spray drying conditions for organic solution advanced spray drying in a closed mode under ultra-high purity nitrogen gas for Lactomorphin and Lactomorphin:Trehalose spray-dried and cospray-dried systems.

Formulations (Powder Composition = Molar Ratio)	Solvent	Outlet Temperature (°C)	Pump Rate (%)
SD-Lactomorphin	Methanol	-	High (100%) *
SD-Lactomorphin	Methanol	31	High (75%)
SD-Lactomorphin	Methanol	47	Med (50%)
SD-Lactomorphin	Methanol	76	Low (25%)
SD-Lactomorphin	Ethanol	52	Low (25%)
SD-Lactomorphin	Isopropanol	51	Low (25%)
co-SD-Lactomorphin:Trehalose = 75:25	Methanol	52	Low (25%)
co-SD-Lactomorphin:Trehalose = 50:50	Methanol	50	Low (25%)
co-SD-Lactomorphin:Trehalose = 25:75	Methanol	44	Low (25%)
SD-Trehalose	Methanol	48	Low (25%)

* No powder formation.

**Table 2:** Particle sizing using image analysis on SEM micrographs (*n* ≥ 100 particles).

Powder Composition (Molar Ratio)	Spray Drying Pump Rate (%)	Solvent	Mean Size (μm)	Size Range (μm)
Raw Lactomorphin	NA	NA	1.51 ± 0.71	0.07–3.54
Raw Trehalose ⁑	NA	NA	NA	NA
SD-Trehalose	Low (25%)	Methanol	0.95 ± 0.28	0.49–1.80
SD-Lactomorphin	Low (25%)	Methanol	0.80 ± 0.25	0.21–1.60
Co-SD-Lactomorphin:Trehalose 25: 75	Low (25%)	Methanol	1.37 ± 0.56	0.11–3.42
Co-SD-Lactomorphin:Trehalose 50: 50	Low (25%)	Methanol	1.33 ± 0.54	0.43–3.86
Co-SD-Lactomorphin:Trehalose 75: 25	Low (25%)	Methanol	1.43 ± 0.37	0.55–2.37
SD-Lactomorphin *	Low (50%)	Methanol	NA	NA
SD-Lactomorphin ⁑	Low (75%)	Methanol	NA	NA
SD-Lactomorphin *	Low (25%)	Ethanol	NA	NA
SD-Lactomorphin *	Low (25%)	Isopropanol	NA	NA

Abbreviations: NA, Not Applicable. * Aggregated particles; ⁑ Chunks-like powder.

**Table 3:** DSC thermal analysis (*n* = 3, mean ± standard deviation).

Sample/Parameter	Raw Lactomorphin	SD-Lactomorphin 75% PR	SD-Lactomorphin 50% PR	SD-Lactomorphin 25% PR	SD-Lactomorphin in EtOH	SD-Lactomorphin in Isop	Raw Trehalose	SD-Trehalose	co-SD-Lactomorphin-Trehalose (25–75)	co-SD-Lactomorphin-Trehalose (50–50)	co-SD-Lactomorphin-Trehalose (75–25)
T_g_ onset (°C)	82.07 ± 1.53	82.59 ± 1.25	87.03 ± 0.64	91.30 ± 2.25	-	98.13 ± 9.84	-	50.88 ± 5.49	70.70 ± 1.35	73.62 ± 6.31	77.43 ±5.27
T_g_ mid (°C)	84.69 ± 0.72	87.18 ± 1.47	90.20 ± 4.36	93.68 ± 0.95	-	100.52 ± 9.50	-	53.14 ± 3.89	73.05 ± 0.63	78.29 ± 6.85	82.13 ± 6.21
T_g_ End (°C)	87.31 ± 0.09	89.99 ± 0.62	93.46 ± 0.61	96.06 ± 2.02	-	104.13 ± 11.62	-	56.27 ± 1.63	79.84 ± 0.71	82.92 ± 7.72	85.92 ± 7.73
∆Cp J/(g·°C)	0.80 ± 0.22	0.72 ± 0.23	0.70 ± 0.0918	0.80 ± 0.15	-	1.20 ± 0.88	-	0.51 ± 0.01	0.43 ± 0.04	0.68 ± 0.02	0.66 ± 0.09
Endotherm1 onset (°C)	175.72 ± 5.98	170.88 ± 3.63	177.66 ± 5.31	180.90 ± 0.44	182.08 ± 2.12	185.54 ± 1.37	94.70 ± 0.08	143.11 ± 5.78	167.96 ± 4.81	176.01 ± 1.59	174.00 ± 0.89
Endotherm1 peak (°C)	175.96 ±6.20	171.50 ±3.24	177.71 ± 5.38	183.10 ± 1.24	182.26 ± 2.00	192.59 ± 8.00	95.85 ± 0.12	144.91 ± 4.91	173.47 ± 11.99	176.46 ± 1.74	174.39 ± 0.95
∆H1 (Enthalpy) J/g	55.43 ± 12.35	43.26 ± 10.80	40.91 ± 2.61	37.25 ± 3.33	2.88 ± 0.52	13.79 ± 0.95	91.92 ± 7.79	1.34 ± 0.10	29.92 ± 8.67	3.68 ± 1.04	3.20 ± 0.43
Endotherm2 onset (°C)	-	-	-	-	233.96 ± 22.56	238.41 ± 17.99	141.32 ± 1.20	187.89 ± 2.16	209.6 5± 8.03	208.65 ± 15.48	214.66 ± 13.72
Endotherm2 peak (°C)	-	-	-	-	247.19 ± 2.1	243.47 ± 14.33	141.01 ± 1.14	196.89 ± 2.70	213.28 ± 7.12	210.53 ± 14.30	215.33 ± 14.78
∆H2 (Enthalpy) J/g	-	-	-	-	35.7 ± 7.08	31.57 ± 4.73	106.90 ± 6.15	56.07 ± 6.21	34.02 ± 9.16	47.73 ±10.30	39.66 ± 8.07
Endotherm3 onset (°C)	-	-	-	-	-	-	196.54 ± 1.10	-	-	-	-
Endotherm3 peak (°C)	-	-	-	-	-	-	204.24 ± 1.10	-	-	-	-
∆H3 (Enthalpy) J/g	-	-	-	-	-	-	118.33 ± 4.17	-	-	-	-
Exotherm (TC) °C	-	-	-	-	150.58 ± 2.28	166.23 ± 0.46	-	110.01 ± 0.95	-	-	-

**Table 4:** Residual water content of SD-Lactomorphin, SD-Trehalose, co-SDLactomorphin:Trehalose powder systems and their raw counterparts analyzed by Karl Fisher coulometric titration (mean ± standard deviation, *n* = 3).

System	Water % (*w/w*)
Raw Lactomorphin	6.07 ± 0.67
Raw Trehalose	8.11 ± 1.96
SD-Trehalose in methanol at 25% PR	4.76 ± 0.54
SD-Lactomorphin in methanol at 25% PR	3.38 ± 0.23
SD-Lactomorphin in methanol at 50% PR	3.96 ± 0.26
SD-Lactomorphin in methanol at 75% PR	4.63 ± 0.70
SD-Trehalose in ethanol at 25% PR	9.67 ± 0.71
SD-Trehalose in isopropanol at 25% PR	4.22 ± 0.15
Co-SD-Lactomorphin:Trehalose 25:75 in methanol at 25% PR	4.35 ± 0.56
Co-SD-Lactomorphin:Trehalose 50:50 in methanol at 25% PR	5.04 ± 1.05
Co-SD-Lactomorphin:Trehalose 75:25 in methanol at 25% PR	5.59 ± 0.38

**Table 5:** Aerosol dispersion performance properties as aerosolized dry microparticulate/nanoparticulate powders including mass median aerodynamic diameter (MMAD), geometric standard deviation (GSD), fine particle fraction (FPF), respirable fraction (RF) and emitted dose (ED) for inhalable microparticle/nanoparticle formulations for inhalable microparticle/nanoparticle formulations of raw Lactomorphin, SD-Lactomorphin, and co-Lactomorphin:Trehalose (mean ± standard deviation, *n* = 3).

Powder Formulation Composition	Emitted Dose	Fine Particle Fraction	Respirable Fraction	MMAD	GSD
(Molar Ratio)	(ED) (%)	(FPF) (%)	(RF) (%)	(μm)	
Raw Lactomorphin	100.33 ± 2.12	27.86 ± 0.94	81.06 ± 0.57	4.29 ± 0.24	4.05 ± 0.74
SD-Trehalose in methanol at 25% PR	88.19 ± 0.40	52.00 ± 0.65	99.05 ± 0.20	1.74 ± 0.04	1.83 ± 0.06
SD-Lactomorphin in methanol at 50% PR	88.98 ± 1.73	52.45 ± 7.47	97.70 ± 0.43	1.66 ± 0.02	1.85 ± 0.10
SD-Lactomorphin in methanol at 75% PR	94.63 ± 0.78	46.15 ± 3.20	94.47 ± 3.62	2.16 ± 0.12	2.02 ± 0.01
SD-Trehalose in ethanol at 25% PR	97.87 ± 15	63.09 ± 2.34	98.76 ± 0.09	1.98 ± 0.07	3.04 ± 0.09
SD-Trehalose in isopropanol at 25% PR	102.29 ± 5.88	66.21 ± 5.17	98.64 ± 0.90	1.64 ± 0.29	3.01 ± 0.27
Co-SD-Lactomorphin:Trehalose 25:75 in methanol at 25% PR	91.24 ± 2.26	50.59 ± 3.88	98.68 ± 0.00	2.21 ± 0.23	2.93 ± 0.96
Co-SD Lactomorphin:Trehalose 50:50 in methanol at 25% PR	88.86 ± 1.13	53.07 ± 2.35	99.15 ± 0.50	1.89 ± 0.04	2.31 ± 0.00
Co-SD Lactomorphin:Trehalose 75:25 in methanol at 25% PR	89.00 ± 0.67	45.92 ± 5.53	95.33 ± 3.99	2.07 ± 0.07	1.84 ± 0.17

**Table 6:** Protomers population% of Lactomorphin at various pHs as calculated by MOE.

pH	Major Protomer%	Minor-1 Protomer%	Minor-2 Protomer%
7.0	60.4	39.3	-
7.5	66.1	33.0	-
8.0	83.4	14.3	-
8.5	87.0	7.0	6.0
9.0	77.3	29.4	-

**Table 7:** Solubility of Lactomorphin in different solvents (mean ± standard deviation, *n* = 3) by experimental and computational methods.

Solubility in:	pH Value	Solubility in mg/mL ± Std dev	Solubility (USP) Definition	Calculated Log S *	MOE Calculated h_logS
Water	8.50 (adjusted)	267.00 ± 6.00	Freely soluble (Fs)	−0.6	−0.6565
PBS	8.50 (adjusted)	570.33 ± 2.00	Freely soluble (Fs)	−0.3	-
NS	8.50 (adjusted)	140.37 ± 0.32	Freely soluble (Fs)	−0.9	-
Methanol	7.27 (measured)	45.67 ± 1.00	Soluble (s)	−1.3	-
Ethanol	7.30 (measured)	18.00 ± 0.00	Sparingly soluble (sps)	−1.7	-

PBS: Phosphate-buffered saline. NS: Normal Saline. * The experimental solubility (S) in mMoleunit.

**Table 8:** Experimental and Computational LogP of Lactomorphin.

Experimental LogP of Lactomorphin at Room Temperature (25 °C) and Physiological Temperature (37 °C) (Mean ± Standard Deviation, *n* = 3)		Computational LogP of Lactomorphin Protomers Exist at pH 8.5 and at Room Temperature (25 °C)	
Temperature (°C)	LogP	Protomer	Computational LogP
		Major Protomer	−2.76
25 °C	−2.40 ± 0.00	Minor-1 Protomer	−2.67
37 °C	−2.33 ± 0.02	Minor-2 Protomer	−7.63
